# Inclusion unlocks the creative potential of gender diversity in teams

**DOI:** 10.1038/s41598-023-39922-9

**Published:** 2023-08-23

**Authors:** Balázs Vedres, Orsolya Vásárhelyi

**Affiliations:** 1 Department of Network and Data Science, Central European University, Vienna, Austria; 2 Oxford Internet Institute, University of Oxford, Oxford, UK; 3 Laboratory for Networks, Technology and Innovation Corvinus Institute for Advanced Studies, Corvinus University of Budapest, Budapest, Hungary; 4 Democracy Institute, Central European University, Budapest, Hungary

**Keywords:** Computational science, Applied mathematics

## Abstract

Several studies have highlighted the potential contribution of gender diversity to creativity, also noted challenges stemming from conflicts and a deficit of trust. Thus, we argue that gender diversity requires inclusion as well to see increased collective creativity. We analyzed teams in 4011 video game projects, recording weighted network data from past collaborations. We developed four measures of inclusion, based on de-segregation, strong ties across genders, and the incorporation of women into the core of the team’s network. We measured creativity by the distinctiveness of game features compared to prior games. Our results show that gender diversity without inclusion does not contribute to creativity, while at maximal inclusion one standard deviation change in diversity results in .04–.09 standard deviation increase in creativity. On the flipside, at maximal inclusion but low diversity (when there is a ‘token’ female team member highly integrated in a male network) we see a negative impact on creativity. Considering the history of game projects in a developer firm, we see that adding diversity first, and developing inclusion later can lead to higher diversity and inclusion, compared to the alternative of recruiting developers with already existing cross-gender ties. This suggests that developer firms should encourage building inclusive collaboration ties in-house.

## Introduction

Groups with diverse members can be engines of creativity. Project teams—small collectives recruited for a defined task—are often used in creative fields, where nonroutine solutions are needed to solve problems^1,2^, and such teams boost returns to resources in innovative organizations^3,4^. There is evidence that teams possess collective intelligence beyond the mean or maximal individual intelligence of team members^5^. It is also often demonstrated, that the collective intelligence and the creative capacity of teams is a function of their cognitive diversity^6^. When team members come from diverse demographic backgrounds and have diverse past experiences, they have a higher openness to divergent thinking^7^, and they are more willing to constructively challenge the status quo^8^.

Gender diversity specifically has been shown to boost collective intelligence^5,9^, and the low proportion of non-dominant genders dampens innovative potential in teams^10,11^. Women, transgender, and gender-nonconforming people (TGNC) are under-represented in STEM fields—especially in computer science and software careers^12–14^, and even if they embark on a career in technology, they are less appreciated and successful, and are more likely to leave at various key stages compared to men^15,16^. Thus it is important to analyze gender diversity in STEM teams to understand how diversity contributes to innovation when females are in minority, and often face discrimination^17^.

Despite a general agreement about the promise of diversity for creativity, studies on how team diversity leads to increased team performance has not reached a clear consensus^18,19^. It is clear that group creativity is not a simple function of individual creativity, but a complex interplay of compositional diversity, internal team structures, and the organizational-cultural environment of the team^20,21^. On one hand diversity itself—while contributing to openness to creative solutions—can contribute to weakened team cohesion, and heightened conflict^22^. On the other hand, the right routines and communication structures within the team can multiply the power of diversity for innovation^4^. Thus we need to consider diversity *together* with inclusion to understand the potential of diversity for collective creativity^23^.

Others have recognized the need to take inclusion into account in understanding collective creativity^24–26^, suggesting that diversity without inclusion can lead to mistrust and a breakdown of communications^27^, preventing a true dialog where diverse approaches to the problem at hand can be explored^28,29^. It had also been shown that the mere increase in the proportion of women in a field will not eliminate their discrimination and marginalization^30^. When diverse teams are well integrated, even if diversity results in conflicts, such conflicts can be beneficial to performance in complex, non-routine tasks^31^. Following these studies, we argue that in teams with a discriminated minority—the case with gender in STEM –, without inclusion diversity will not have a positive impact on collective creativity, as various perspectives that diverse participants bring to the team would not have a chance to be contrasted and utilized.

### Gender diversity and collaboration in video game development

Video game development is by far the largest entertainment industry today, as it overtook movies and music in terms of gross revenues in 2003, and, by 2009, it became larger than movies and music combined^32^. Video game development is a field that prizes creativity and distinctiveness^33,34^, but it is a male-dominated field, where only about 17% of developers were female in 2010, and about 20% of them are female today^35^. The typical content of video games is also decidedly male, as only about 13% of all characters depicted in video games are female^36^. Video game content often encodes gender stereotypes and role expectations^37^. Gender inequalities have been demonstrated to influence how players achieve and experience success^38^. Such fields with low proportion of female participants also suffer from strong prejudice and discrimination against women^30^, thus if we are able to show creative advantage to gender diversity in this strongly male dominated field, it would serve as a strong evidence for the power of gender diversity.

We analyze teams in the video game industry from 1994 to 2009. While there is a rich literature on how team composition and network ties influence performance in gameplay^39,40^, we focus on team composition of developers creating games^34,35^. We collected data about 8617 unique video games, relying on a website that catalogues video games via crowdsourcing and covers over 230,000 games from more than 60 countries.—MobyGames.com. Our database contains information on each game’s developer teams, critic’s reviews, and stylistic elements such as genres, perspective (e.g., first-person shooter, role-playing) and the platforms it can be played on (e.g., PlayStation, Nintendo Switch, etc.). We also record each game’s developer studio, publishing house, market where it is available, and the year of the first release. We added data on global sales in thousands of units sold from https://www.vgchartz.com. For our analysis we only considered games which were published between 1993 and 2009, and had less than 2000 connection among team members, had at least one female team member, and less than 50% of team members gender could have been inferred. We excluded all re-released and mobile games. Our resulting database contains 4,011 video games. (For more details on filtering our database see Tables S1, S2 in Supplementary Information.)

Since our database goes back to the very beginnings of the video game industry, we can infer every developer’s full career path, by connecting disambiguated developer IDs with the IDs of games they had worked on in a consecutive order. We measure weighted collaborative ties between developers as the number of prior joint participations in game development projects, following others who have analyzed collaboration in co-authorship^41^, movies^42^, musicals^43^, video game development^34^, or jazz music^44^. These approaches take a bipartite graph of person-to-event affiliations (affiliations to papers published, movies, games, or albums released), and analyze the person-to-person projection, an undirected weighted graph, where ${w}_{ij}=\sum {a}_{ik}{a}_{jk}$, if $k$ is a shared affiliation for $i$ and $j$ at time $t-1$ that predates time $t$ of the focal event analyzed. While we follow widespread practice by defining our weighted collaboration network^11,34,43,45,46^, we also acknowledge that there are several relevant dimensions of collaboration (for example, communication ties, collaborations outsize our game dataset, or social ties among developers) that we do not have data about.

### Measuring inclusion

Inclusion in a work-team context can be defined as actively engaging with team members across differences: inclusion means connecting in a way such that diversity becomes a resource, rather than merely being a challenge^25^. We cannot speak of inclusion, when differing team members are excluded from meaningful contact and collaboration, either by isolating individuals that are seen to be different, or allowing the team to fragment into homophilous subgroups. Inclusion is also absent when team members are fully assimilated, such that by connecting to others their diversity becomes muted and irrelevant in collaborations^47^.

There is no consensus about how inclusion should be measured. A wide range of measures were proposed, that include dimensions of individual or group experiences, leadership, norms and values^25^, influence on decisions, and access to resources^48^, a sense of belonging, and authenticity^49^, organizational support, and tolerance towards uniqueness^50^. These measures require reactive data collection techniques (like surveys or interview methods), and are not scalable to large observational data.

In this article we focus on the relational aspects of inclusion, and we rely on weighted graph measures of how well gender minority members in the team are connected into the collaboration network, as evidenced by past projects. We build on past research that developed related measures conceptualized as network heterogeneity^51^, or the co-presence of incumbency and network diversity^46^. We develop a range of measures for inclusion, from a minimal level—lack of segregation along gender—to a stronger level—the presence of gender minority in the network core of the team.

**Figure 1:**
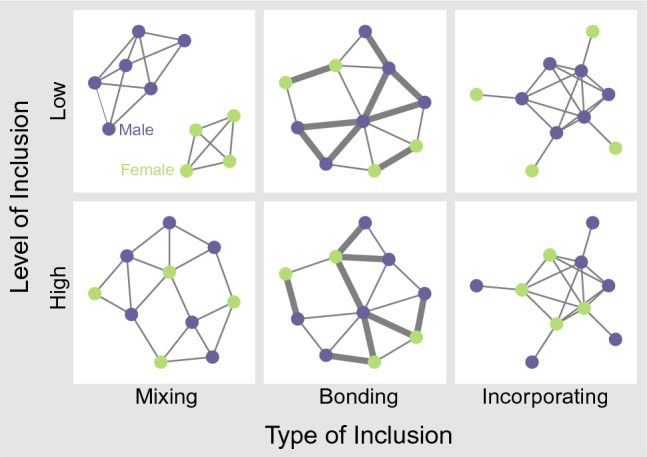
Illustrations of low and high inclusion along our three measures.

We define three dimensions of inclusion, Fig. 1 illustrates these measures examples of weighted collaboration graphs, where the number of nodes and the proportion of genders are kept constant. Our first measure of inclusion is *mixing*: the lack of segmentation by attributes in the team. If team members are segregated, the team cannot benefit from exchanges across gender lines. Limiting collaborative connections that would allow for negotiating diverse perspectives, essential for innovation^52^. When gender underlie subgroup formation, team identity erodes, and the salience of gender identity increases often to the extent of gender conflict^53^. Network fragmentation along attributes can be measured by assortativity, the over-representation of ties within categories^54^.

Our second measure of inclusion is *bonding*, that captures the strength of ties across gender categories. Strong ties are seen to be vehicles of trust^55^, and they offer high-bandwidth interpersonal channels that are crucial in innovative contexts, when the information environment is complex, and updates frequently^56^. The stronger the ties in mixed gender dyads, the broader the social bandwidth a team can rely on to develop novel solutions.

Our third measure of inclusion is *incorporating*: the proportion of female team members in the core of the team’s network. Being in the core opens access to informal leadership, and thus offers the opportunity for women to have a say in decision making^47^. Women in leadership positions tend to encourage participation, and facilitate broader information sharing^57^, and encourage innovation and risk-taking^58^. Our measure of *combined inclusion* is then the product of the three raw measures, representing co-occurrence of various forms of inclusion. (See Quantitative Measures in Materials and Methods for formulas of inclusion metrics.)

### Measuring creativity and diversity

We adopt a creativity measure—*distinctiveness*—defined for the video game context, which captures how unique the combination of stylistic elements was for a given game. For each game we record the presence or absence of 105 stylistic elements (recording features of perspective, gameplay, genre, or sound). We then compare games by the uniqueness of their combination of elements. The measure compares the combination of stylistic elements of a focal game to all games released in the preceding time window, using cosine distance^34^, where a high distance indicates a game that included a unique combination of features. (We tested the robustness of the results with 1, 3, 5, and 7 year windows, and with conceptually similar novelty metrics^11,59^, and present findings with five year window in the main text following^34^). Gender diversity is quantified by Blau’s Heterogeneity Index, which reaches its maximum, 1, when the proportion of genders are equal (50% in this case) and has a minimum of 0 when the team is composed of only one gender. (See Quantitative Measures in Materials and Methods for formulas.)

## Results

### Predicting distinctiveness by diversity and inclusion

**Figure 2:**
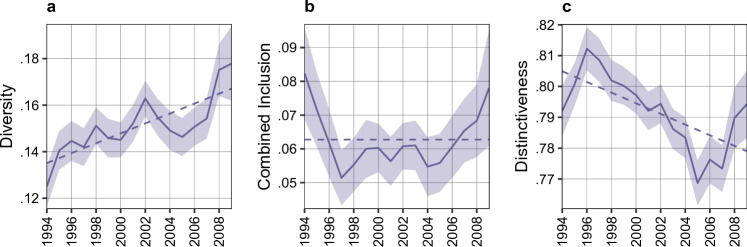
Gender Diversity, Inclusion and Distinctiveness over time. Solid lines indicate the mean with a 95% CI. Dashed lines show OLS trendlines. Panel a: Diversity measured by the mean proportion of female developers within game projects, and it is measured on the full sample (8617 games). Panel b: Mean combined inclusion within game projects, measured on the sample of games with at least one female developer (4011 games). Panel c: Mean distinctiveness (compared to games in the preceding 5 year window) within game projects.

Gender diversity in video game development is low, albeit increasing, while there is no increase in inclusion. The proportion of female game developers is 0.15 across all teams in our dataset: less than the proportion of females in STEM and computer programming^60,61^, that is around 20%. (See Gender Inferring in Materials and Methods.) As shown on Fig. 2 panel a, the female proportion of game developers had been slowly increasing from 0.12 in 1994 to 0.18 in 2009. There is no comparable increase in inclusion (as shown on Fig. 2 panel b), as the combined inclusion index hovers around a mean of 0.06 without a significant trend. Considering trends in creativity (game distinctiveness), we see an early peak in 1996, and a subsequent downward trend, as industry practices and conventions solidify (see Fig. 2, panel c). However, the overall level of creativity is quite high.

We predict distinctiveness by diversity and inclusion via LOESS and OLS regression models where the unit of analysis is a game. We ran separate models for each inclusion. In each full model we controlled for multiple variables representing potential alternative explanations for the association between diversity × inclusion and distinctiveness: *Team size* is measured as the number of team members involved in the game production. Larger teams are typically assembled by more established developer firms, therefore more likely to have bigger networks and employ more women, and also have more leeway to experiment with novel game ideas^8^. *Ratio of center* measures the proportion of developers in the team network who belong to the center. Flatter organizations (with a larger center) are also more likely to have women in the center just by chance, while they are also likely to be more creative^62^. *Number of Newbies* measures the number of team members with no prior experience in game development (based on our database). Newbies might bring novel ideas to the team, but we have no data on their level of inclusion. We also recorded the *Number of star developers*, those who have received a Game Developers Choice Award. Awardees are typically male and can afford to develop more distinctive games. *Game tenure* captures the experience level of a team, measured as the average number of games team members have produced prior to the year of production of the given game. *Single-Firm Production* is a dummy variable, which is 1 if the publisher and the developer company is the same entity, otherwise 0. Such integrated companies could make a more immediate connection between game design and consumer preferences, moving faster to distinctive solutions. We controlled for the *platforms* the game was developed for, because certain genres and platforms can be more popular than others—both with consumers and developers. We also controlled for temporal trends, with the t *year of release* and the *number of countries* the game was released at.

**Figure 3:**
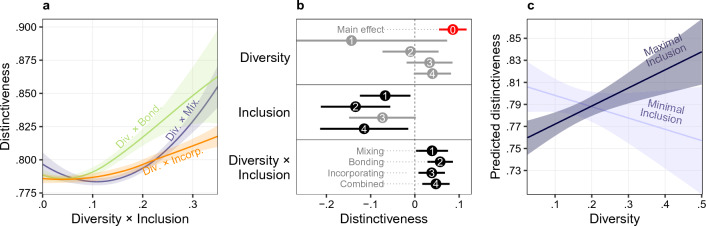
Distinctiveness as a function of the interaction of diversity and inclusion. Panel a: LOESS estimates of distinctiveness by the product of diversity and three measures of inclusion with 95% CI; separate lines are shown with abbreviated labels for diversity × mixing, diversity × bonding, and diversity × incorporating. Panel b: OLS point estimates with clustered standard errors for developer firm of standardized distinctiveness with 95% CI, for: gender diversity, variables of inclusion, and their interactions with gender diversity. Markers are numbered according to OLS models; coefficients represent one SD change in distinctiveness as a result of one SD change in independent variables. Point estimate for the main effect of gender diversity without entering inclusion is shown as Model 0, in red. Non-significant estimates at *p* < .05 are shown in gray. Panel c: Predicted distinctiveness by gender diversity at minimal and maximal levels of Mixing Inclusion with 95% CI, keeping all controls at their means. The figure shows model predictions for synthetic data, where we can compare the predicted distinctiveness of synthetic game projects as we manipulate diversity, and keep inclusion either at the minimum (0) or at the maximum (1)^64,65^.

As expected by the literature^5,9–11,63^, we find that gender diversity is positively related to creativity in video game projects, even without considering inclusion. Considering games as units of analysis, one standard deviation increase in gender diversity results in 0.09 (95% CI 0.06; 0.12) standard deviation increase in creativity (as shown on Fig. 3 Panel b).

**Figure 4:**
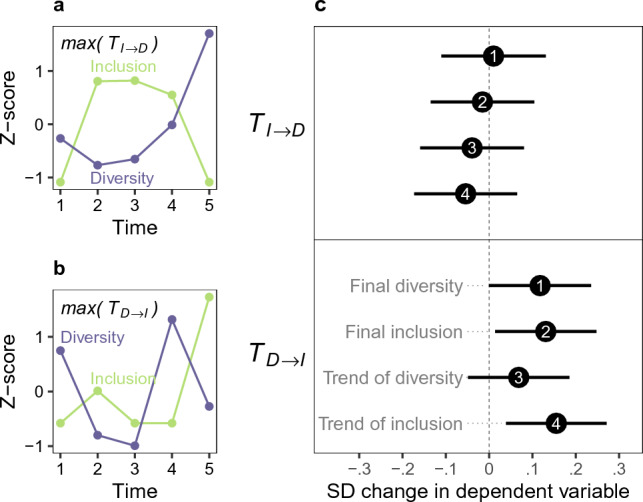
Developer firm-level relationship between the times series of diversity and inclusion. Panel a: Example of an inclusion driven processes of length five where transfer entropy ${T}_{I\to D}=\mathrm{max}{T}_{I\to D}$. Panel b: Example of a diversity driven processes of length five where transfer entropy ${T}_{D\to I}=\mathrm{max}{T}_{D\to I}$. Panel c: Point estimates for predicted diversity and inclusion (final value and trend) by the nature of firm-level processes: transfer entropy of inclusion to diversity, and transfer entropy from diversity to inclusion, when these transfer entropies are simultaneously entered in an OLS prediction. Horizontal axis shows predicted SD change in outcome when independent variables increase by one SD.

We found a positive overall relationship between distinctiveness and the interaction (product) of diversity and inclusion, as the LOESS curves show on Fig. 3 Panel a. Taking control variables into account in OLS models, Fig. 3. Panel b. shows points estimates of standardized gender diversity, forms of inclusion, and the interaction of gender diversity and inclusion. Our results hold if different robustness checks are applied (1) distinctiveness is calculated on different time windows (1, 3, 7), (2) creativity is conceptualized as novelty followed by^11^, and (3) if models are applied on different subsets of database suggested by^66^(See Creativity Prediction Model Tables in SI Tables S4–S27., and Prediction Results on SI Figs. S5 and S6.)

We found that once we take inclusion into account, gender diversity without inclusion does not contribute to group creativity. We also found that inclusion without diversity (with the minimal diversity possible) does not contribute to group creativity. The main effect of inclusion is mostly negative: two of our three measures (mixing and bonding) show significant negative coefficients (mixing: − 0.07, 95% CI − 0.12; − 0.01; bonding: − 0.13, 95% CI − 0.21; − 0.06). Our results hold if we control for developer firm level heterogeneity (with random or fixed effects for developer firm intercepts), or when we aggregate the data to the developer firm level. (See firm-level point estimates in Fig. S2, panel d in Supplementary Information.)

These estimates indicate that inclusion without gender diversity does not help creativity. Of course, inclusion is not interpretable for zero diversity; these estimates indicate that increasing inclusion at minimal levels of diversity will not help creativity. It is likely that high inclusion at low levels of diversity leads to assimilation and tokenism that was previously shown to nullify creative benefits to diversity^47^.

Creativity in game development benefits from gender diversity *with* inclusion only. Game developer teams should include female collaborators, *and also* integrate them with the rest of the team to see a boost in creativity. Figure 3 Panel c shows the predictions in the case of mixing inclusion. (Marginal predictions for all four inclusion variables are in the Supplement Fig. S3.) Across all four inclusion measures our predictions indicate that video game developer teams cannot increase their creativity at any level of gender diversity if female developers are not included (their inclusion measures equal zero): Adding “newbie” female team members without prior collaborative ties to male team members will not contribute to increased creativity in the first instance. In contrast to predictions with minimal inclusion, at maximal inclusion an increase in gender diversity leads to increase in creativity. If a team moves from the lowest gender diversity in our dataset to the highest, while maintaining maximal inclusion, it can boost creativity (game distinctiveness) by 22% (considering our combined inclusion measure).

Of our controls we see a consistent negative coefficient for team size and the number of countries a game was released: larger companies are available on more markets and have larger teams that produce lower distinctiveness, in line with previous analyses of video game team data^34^.

### Firm-level processes that lead to diversity and inclusion

How can firms boost diversity and inclusion? Our results indicate that a firm with only male developers would find it difficult to realize creativity benefits from adding female developers incrementally: Considering firms that include female developers for the first time after working with only male developers (there were 306 such firms in our dataset of the complete set of 1354 firms), we do not see any increase in creativity. The distinctiveness score of the first game with any females is even slightly (but not significantly) lower than the preceding game with males only. What our predictions indicate is that developer firms need to reach diversity in the top quartile to start seeing benefits from gender diversity, when female developers are also included in the team’s network. Furthermore, firms with low diversity would experience a decrease in creativity as they increase inclusion in the team. This presents a barrier to increasing diversity in video game developer firms. Nevertheless, several developer firms did successfully increase diversity and inclusion, thus we need to understand processes that can lead to higher levels of diversity and inclusion, despite the lack of early benefits.

We turn to analyze histories of game developer firms, to understand if intervention in diversity, or intervention in inclusion is the more promising avenue to overcome barriers in developer firms. In the first case, if boosting diversity is the key to advancing both diversity and inclusion, firms can add female developers to their teams, and then subsequently see an increase in inclusion, when female developers build ties to male developers in repeated game projects. In the second case, if firms can intervene by adding inclusion, the key is to hire subsets of developers with gender diversity *and* pre-existing ties between female and male team members. In this case, inclusion is not “home grown”, but rather a function of clustered migration of developers among firms. This process was recently described as the “trojan horse” mechanism^67^, driven by a sequence of clustered migration of individuals who have prior collaborative ties between them.

To capture the primary drivers of firm-level processes, we use transfer entropy, a measure that captures the amount of information that values in one time series have about subsequent values of another time series^68,69^. We measure transfer entropy between two processes: the developer firm-level times series of diversity and inclusion, in both directions. We enter the resulting variables, transfer entropy ${T}_{D\to I}$ and ${T}_{I\to D}$ in an OLS model that predicts the final diversity and inclusion (the last values in time that we see for a given firm in our dataset), and the trends of diversity and inclusion at the developer firm level. Figure 4 Panels a and b show two examples, the one Panel a where inclusion predicts diversity the most, and a second on Panel b where diversity predicts inclusion the most.

Figure 4 Panel c shows point estimates predicting firm-level diversity and inclusion. We found that boosting diversity and inclusion seems to be a product of a diversity-driven process, where changes in diversity result in changes in inclusion. This process in practice can be conceptualized as the hiring of female developers regardless of their prior histories of collaboration with other team members, and subsequently adding inclusion by facilitating repeated collaborations between female and male developers—a form of “home grown” inclusion. The reverse direction of temporal influence between processes does not seem to lead to increased diversity or inclusion: When firms add inclusion first (and diversity is a result of this subsequently), we should expect no measurable advantage in increased diversity or inclusion. In practice such a process would mean hiring dyads of female and male developers with pre-existing collaboration ties, which we could label as “acquired inclusion”.

This indicates that developer firms should not hesitate to add novice female developers to their teams—even if they cannot expect immediate creativity benefits in the team with female developers without inclusion, as female developers would not yet have cross-gender collaborative ties. Female developers will accumulate collaborative ties, and thus achieve inclusion subsequently, and the team can expect to see a boost in creativity.

## Discussion

As others have already found, we have also shown that gender diversity is a predictor of creativity^9–11,63^: adding females to a team in a male-dominated industry like video game development contributes to the distinctiveness of the final product. However, it is not enough to add female developers to a team, but female team members should also be connected into the collaborative network of the team. Without inclusion we see no creative advantages in diverse teams. Gender diversity interacts with inclusion in a way that diversity without inclusion does not bring any advantages in creativity, regardless of the extent of diversity.

Our results indicate that organizations should pay attention to inclusion as well, not only to diversity. There is a rich literature on inclusion stressing the importance of integrating employees and team members with diverse attributes^25,48–50^, but systematic and large scale measurement for inclusion, diversity, and creativity had not been developed in conjunction. We operationalize inclusion using the network of past collaborations, developing three diverse metrics that all support the same conclusion: gender diversity without inclusion does not lead to benefits in creativity.

At the same time, we also see evidence that tokenism—the minimal presence of a gender minority—is not effective. When an all-male team adds one female member only, the team can expect no creativity benefits. In fact, when we observe developer firms with exclusively male teams in their history adding a female developer for the first time, there is a slight decrease in creativity. Our findings underscore prior findings about the limits of tokenism^70,71^: when gender diversity is low, inclusion acts more as assimilation that possibly silences the creative potential in diversity. One female team member closely linked with others in the team will not be able to voice and represent a diverse perspective in a male-dominated industry.

Our results point to several reasons why organizations would face difficulties increasing gender diversity. First, as a developer firm would be looking to increase diversity from zero, or very low levels, it would not see early benefits in increased creativity. Organizations need to add and also include in their collaborative network a relatively high proportion of female developers (about 23%—significantly higher than the industry average of 19%) to start seeing creativity benefits. This delayed onset of benefits is likely a contributor to the sustained marginalization of female developers in the field, reinforcing beliefs in the benefits of male-skewed (or all-male) team composition. Second, even if a video game developer firm is successful in achieving higher creativity via diversity and inclusion, the payoffs in terms of success will be hampered by gatekeepers that review video games. Games made with higher gender diversity face a higher probability of being ignored (not being reviewed at all). This selectivity seems to prevent games made with higher gender diversity from reaching higher review scores. While there is a positive link between gender diversity × inclusion, and distinctiveness, this does not translate into a positive link between gender diversity × inclusion and success. (See Diversity, inclusion, and forms of success in SI Fig. S8).

Games made with higher female participation are disadvantaged in reviews: the probability that a game will be reviewed at all, sharply declines as the proportion of female developers increases. It seems that reviewing is a male-dominated activity, that can possibly lead to a dis-preference towards games made with higher female participation, as the named list of developers is also visible to reviewers. (See the name and the inferred gender of publicly available employees including game reviewers at Kotaku, Gameinformer and Eurogamer at SI Table 29–31) Beyond being disadvantaged in the chances to get a review, games where diversity × inclusion is higher we see a significantly lower review score, even if we keep distinctiveness and other relevant features of the team, game, and company constant. However, once we control for selection bias—the fact that review scores are not missing at random, but exclusion form reviewing is a strong function of gender diversity—we do not see a disadvantage for diversity × inclusion in term of review score. This suggests that if we can eliminate discrimination against gender diversity in the decision to review games, the creativity benefit that stems from diversity × inclusion would translate into a higher review score, hence distinctiveness is a significant positive predictor of review score. (See Heckman Selection Model and OLS model in SI Table S28).

Firms might be tempted to hire subsets of developers with diverse genders *and also* with prior links of inclusion already established among them—taking a wholesale approach to jump-start a diverse and inclusive creative team. However, we found evidence that it is more beneficial to take a stepwise approach: Firms should first increase diversity, and then inclusion. By our results, organizations should aim to recruit novice, unconnected female collaborators, and then increase their inclusion by employing these novice female team members in repeated projects. The alternative approach of recruiting diverse team members already with a history of cross-gender collaborations does not lead to a sustained increase in diversity and inclusion.

### Limitations

Limitations of our study chiefly relate to the definition and measurement of diversity and collaboration. Gender identity is not binary, however such personal information could be only analyzed if self-claimed gender identity is provided, therefore we could not incorporate non-binary gender into this study. We are also aware of the limitations and the potential biases of name-based inferring methods, such algorithms perform better on Western-names^72^. Therefore we inferred the name-based ethnicity of developers using an increasingly popular web solution the Ethnea api^73^. To quantify the impact of unknown filtering we compared the ratio of developers with Western names within our final dataset, and before filtering teams out with more than 50% unknowns. We found no significant difference between the distribution of Ratio of Western origin team members (Mann–Whitney Statistics = 11,060,581.000, *p* = 0.482).

To account for the potential bias that the presence of unknowns within teams implies we performed robustness checks and found even if 50% of unknowns are male the positive interaction of gender diversity and inclusion persists in the case of combined index and bonding but mixing and incorporating are more sensitive to such bias. (For further details see Impact of Gender Robustness on Modelling in SI, and Figs. S4 and S5.)

Our measurement of past collaboration was restricted to collaborations within the population of games in our dataset, thus we have no data about past collaborations in game projects not in the database, or projects in other industries. To fully capture inclusion, we would also need to have multiplex network data about communication and other relevant on-project relationships, as well as a subjective sense of acceptance. Our measure of diversity did not take dimensions beyond gender into account, while in collaborative settings complex intersectional diversity is at play.

## Methods

### Gender inferring

Similarly, to film credits, Moby Games lists each team member’s full name and task in the production (imaging, scripting, design, music, etc.). To infer team members’ gender, we relied on developers’ full names, and adopt a commonly used first-name based gender inferring method^16^. Specifically, our method is relying on 2016 US baby game database, published by the US Social Security Administration annually. (SSA 2016). Our algorithm splits names in credit lists into first and last names, then checks whether the given first name is in our database and for which gender. Since some names are used for both males and females each name has a probability of being male based on the fraction of times when the given first name was assigned to a male baby. To be very certain of the result of the gender inferring method, we only label those as male whose probability is higher or equal to 0.9 and as female is lower or equal to 0.1. Names that have only initials, or their probability is between 0.1 < *p* < 0.9 are labelled as unknowns. The method that we selected is optimized for high precision, where names with high probability for being unisex are labelled as unknowns. Our gender inferring yielded 19% female, 63% male and 18% unknowns. (For further details on the accuracy of gender inferring see Accuracy of Gender Inferring, Fig. S1, Table S3 in Supplementary Information.)

### Quantitative measures

Dependent Variable: We measure *creativity* by adopting De Vann et al.’s distinctiveness metric, which compares the combination of each game’s stylistic elements to all games released in the preceding 5 years and compute and average distance (1- cosine similarity) between them^34^. (See SI List of stylistic elements used to quantify Distinctiveness and Novelty) Since we do not know the exact publish date of a very game, we did not compare games published within the same to avoid temporal aversion.

Cosine Distance ${\mathrm{d}}_{\mathrm{i},\mathrm{j}}$ is calculated (1) by comparing the vectors of stylistic elements of all game *i* with all other game *j,* the following:

$$d_{{i,j}} = 1 - \left\lceil {\mathop \sum \nolimits_{{k = 1}}^{K} g_{{ik}} g_{{jk}} /\left( {\mathop \sum \nolimits_{{k = 1}}^{K} g_{{ik}}^{2} } \right)^{{1/2}} \left( {\mathop \sum \nolimits_{{k = 1}}^{K} g_{{jk}}^{2} } \right)^{{1/2}} } \right\rceil$$

where *g*_*ik*_ is 1/*K* if a given stylistic element *k* was used in game *i* and 0 otherwise. Then the resulting similarity is subtracted from 1. (2) Finally we normalize these game-pair distances for all games (1, 2,…, j) published in the proceeding 5 years, as the following:

$$Creativity=\sum_{j=1,j\ne i}^{N}{d}_{ij}/N$$

Independent Variables—Gender Diversity and Team cohesion metrics.

#### Gender Diversity: Blau’s index

We use Blau’s Heterogeneity Index as our measure of diversity. It is calculated as ${H}_{B}=1-\sum {P}_{i}^{2}$, where ${P}_{i}$ is the ratio of group members in category *i* (male or female). Therefore, the female-male ratio is 50–50% the Blau’s Index is 1, and when a team is composed only by one gender group is 0.

We measured inclusion in four ways by using network-based segregation metrics:

#### Mixing as reversed assortativity

Assortativity Coefficient developed by Mark Newman^74^ measures the similarity of connections in the graph with respect to the given attribute. It has been widely used to measure homophily in various (social) networks: such as sexual contacts and marriage matching^74,75^, demographics on Facebook^76^, book recommendation networks^77^ or the research interests of scientists who follow each other on twitter^78^. The Assortativity Coefficient, r is calculated as the Pearson correlation coefficient of degrees between pairs of nodes, formally $r=\frac{Tr(M)-\sum {M}^{2}}{1-\sum {M}^{2}}$, where *M* is the mixing matrix (joint probability) of the two genders, and *Tr(M)* is the trace (sum of elements in the diagonal) of matrix *M*. *r* = *0* is where the network is perfectly disassortative, meaning that every edge connects a node to a different type, while *r* = *1* means perfect assortativity, when the network is fully segregated, such that nodes from type i do not connect to nodes to type j. We quantify reversed assortativity by subtracting it from one and normalizing it: $Mixing=|1-r|/max(|1-r|)$. Large values of Mixing mean high inclusion—team members are mixed by gender, and low values indicate gender segregation.

One of the beneficial attributes of assortativity coefficient while measuring segregation is that this metric is insensitive to the number of isolated nodes within the network^79^. Because our collaboration networks are based on previous shared collaborations we have a higher number of isolated group members, which we should not consider while analyzing the network structure.

#### Bonding as the ratio of weighted cross-gender ties

More frequent shared project experience indicates more intense relationship among team members, which can be a proxy for higher inclusion. Women have been shown to strive and feel more included in workplaces where they could develop stronger ties^80^. Stronger ties were also shown to be beneficial to transfer complex knowledge^81,82^ and solve complex problems^83^. Therefore, our second metric quantifies gendered inclusion as the total number of times men and women worked together in previously divided by the total number of shared working experience of team: $Bonding=\sum {W}_{FM}/\sum_{i}{W}_{i}$, where $\sum {W}_{FM}$ is the sum of weights that connect different gender groups, and $\sum_{i}{W}_{i}$ is the sum of all weights within the network.

#### Incorporating as the ratio of women in the graph center

Our third inclusion metric captures how central women’s position within the team network, specifically the ratio of women within the collaboration network’s center. Network center is defined as the Jordan center of a graph, which is a set of nodes where eccentricity is equal to graphs’ radius. The eccentricity $\epsilon (n)$ of a node $n$ measures how far a node is from the furthest node in the graph. Formally $\epsilon (n)=\underset{u\in N}{\mathrm{max}}(n,u)$. The radius $r$ of a graph is the minimum eccentricity of any node, formally $r=min(\epsilon (n))=\underset{n\in N}{\mathrm{min}}\underset{u\in N}{\mathrm{max}} (n,u)$ To measure which team members belong to the center we used “Python 3. NetworkX Center Distance Metric.” Finally we take the natural logarithm of the ratio of women in the center to ensure a better distribution, therefore calculated as $ncorporating=log(\frac{{N}_{w\in C}}{{N}_{w}})$, where ${N}_{w\in C}$ is the number of women in the center ${N}_{w}$ $\mathrm{is the number of women in the team}.$

#### Combined inclusion

Our fourth measure of inclusion is the combination of the first three, as a product of the three measures of inclusion: mixing, bonding, and incorporating.

#### Time series analysis

We have filtered our data to include games from developer firms that had at least four games in the dataset. This resulted in a dataset with 2418 games from 308 developer firms, filtered from the original dataset of 4011 games from 1354 firms. Distributions of key variables (creativity, diversity, and combined inclusion) in the filtered dataset did not differ from the full dataset (with Kolmogorov–Smirnov test p values of 0.99, 0.65, and 0.83 respectively), and the means of these variables were not significantly different either (with Wilcoxon rank sum test p-values of 0.57, 0.22, and 0.83 respectively). We recorded the diversity and combined inclusion scores for these games, and we calculated transfer entropy from diversity to inclusion, and from inclusion to diversity as ${T}_{X\to Y}=S\left({Y}_{t}|{Y}_{t-1:t-L}\right)-S({Y}_{t}|{Y}_{t-1:t-L},{X}_{t-1:t-L})$, where $S(Y)$ is the Shannon entropy of $Y$.

Since time resolution for the publication date for games in annual, we had several games within a developer firm that were from the same year. For these games with tied dates we have used random sorting, and re-calculated transfer entropies. We used 500 random sortings of ties for all temporal sequences. We then calculated the mean transfer entropy scores of these 500 sequences for each developer firm game sequence.

### Supplementary Information

Supplementary Information.

## Data Availability

Dataset used for analysis is publicly available at that link: https://github.com/velf/moby_data.
