# Supplementary material for: Inclusion unlocks the creative potential of gender diversity in teams
Source: Sci Rep. 2023 Aug 23;13:13757. doi: 10.1038/s41598-023-39922-9 (PMC10447557; doi:10.1038/s41598-023-39922-9)
Supplement: Supplementary file 1 — Supplementary Information. [file 41598_2023_39922_MOESM1_ESM.docx]

# Supplementary Information

Inclusion unlocks the creative potential of gender diversity in teams

Balázs Vedres, Orsolya Vásárhelyi

**Content**

**Supplementary Text**

**Figs. S1 to S5**

**Tables S1 to S12**

**Data S1**

**Code S1**

***Accuracy of Gender Inferring***

To quantify the error in our algorithm we randomly selected 200-200 team members whose gender was inferred as male, female and unknown and manually inferred their gender. Since these people re professionals, we look-up their profiles on LinkedIn and labelled them based on their pictures, bio, and recommendations (if the text used gendered language aka, He or She). If we could not find any LinkedIn user with the same name or based on the picture or the text it was not possible to decide whether the given individual is a male or female, we labelled them as unknown.

Furthermore, we ran another widely used gender inferring method on our database gender-guesser (<https://pypi.org/project/gender-guesser/>) to compare how well our method performed. Figure S1. shows the accuracy of the used gender inferring method and the Gender Guesser Python package in comparison with the manually created baseline. Precision measures how many contributors were assigned to the correct gender label according to our baseline. Recall measures how many of the contributors were correctly identified. F-score takes the harmonic average of these two metrics. Our method has a higher precision for females and males, but lower recall for male. Table S3. Shows that our method is also better identifying unknowns than the Gender-Guesser default python package, but worse in identifying male contributors.

**Impact of Gender Robustness on Modelling**

To account for the potential bias that inferred gender could introduce to results, we adopt various robustness checks. Although the precision of our name-based gender inferring method was nearly perfect for men and women, we accounted much lower precision for unknowns (50%). Although we excluded teams with more than 50% of unknowns from our analysis, there is still bias that team members with unknown gender can add to our results. Statistics on female representation in the video game industry indicate that most of the unknowns are more likely to be male. Therefore, we randomly select 25 or 50 percent of unknown gendered team members in each game and re-label them as males and re-calculate all diversity and inclusion metrics. We repeat this process 100 times and take the average of the resulting inclusion metrics for each game. (Figure S3, in SI shows the distribution of newly calculated gender diversity and inclusion metrics with 25 and 50 percent of relabeled data compared to original data). Then we rerun game-level OLS models to predict games’ distinctiveness based on the 25 and 50 percent relabeled diversity and inclusion metrics. The interaction between gender diversity and bonding stays significant even if 50 percent of unknown gendered team members are labelled as males. Similarly, to bonding, combined inclusion’s interaction with gender diversity is robust to gender relabeling, while mixing and incorporating lose their significance if at least 25 percent of unknowns turn out to be male. (See the Distribution of gender swapped diversity and inclusion metrics at Figure S4 and Point estimates of distinctiveness based on OLS models ran on relabelled gender data at Figure S5)

**List of stylistic elements used to quantify Distinctiveness and Novelty**

'Action', 'Puzzle', 'Adventure', 'Strategy', 'Tactics', 'DLC', 'Add-on', 'Simulation', 'Compilation', 'Special Edition', 'Role-Playing (RPG)', 'Educational', 'Racing', 'Driving', 'Sports', '1st-person', 'Side view', 'Top-down', '3rd-person [DEPRECATED]', "Bird's-eye view", 'Behind view', 'Text-based', 'Spreadsheet', '3rd-person', 'Audio game', 'Fixed', 'Flip-screen', 'Free-roaming camera', '2D scrolling', 'Isometric', 'Cinematic camera', 'Shooter', 'Platform', 'Puzzle-solving', 'Interactive Fiction', 'Text Adventure', 'City Building', 'Construction Simulation', 'Managerial', 'Business Simulation', 'Japanese-style RPG (JRPG)', "Beat 'em up", 'Brawler', 'Arcade', 'Hidden object', 'Board Game', 'Wargame', '4X', 'Action RPG', 'Survival Horror', 'Stealth', 'Visual Novel', 'Metroidvania', 'Fighting', 'Cards', 'Tiles', 'RPG Elements', 'Paddle', 'Pong', 'Chess', 'Casino', 'Game Show', 'Trivia', 'Quiz', 'Word Construction', 'Graphic Adventure', 'Virtual World', 'Sandbox', 'Open World', 'Pinball', 'Music', 'Rhythm', 'Rail Shooter', 'Mini-Games', 'Vehicle Simulator', 'Tactical RPG', 'Mental training', 'Falling Block Puzzle', 'Vehicular Combat Simulator', 'Martial Arts', 'Timed Input', 'Tower Defense', 'Hunting', 'Tile Matching Puzzle', 'Party Game', 'Tricks', 'Stunts', 'Trading', 'Collectible Card', 'Dating Simulation', 'Roguelike', 'Hack and Slash', 'Life', 'Social Simulation', 'Time Management', 'Quick Time Events (QTEs)', 'Artillery', 'Japanese-style Adventure', 'Interactive Book', 'Tactical Shooter', 'Direct Control', 'Text Parser', 'Menu Structures', 'Motion Control', 'Point and Select', 'Multiple Units', 'Characters Control', 'Voice Control', 'Detective', 'Mystery', 'Spy', 'Espionage', 'Comedy', 'War', 'Horror', 'Romance', 'Adult', 'Healthcare', 'Crime', 'Thriller', 'Survival'

Figure S1.


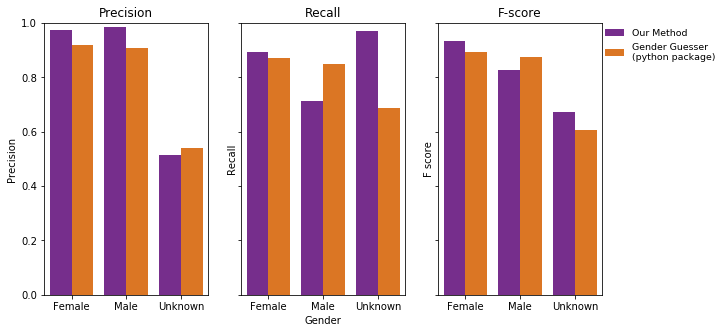


**Gender Inferring Accuracy.** Precision, Recall and F-score of our gender inferring method and the Gender Guesser Python package in comparison with the manually created baseline. Precision measures how many contributors were assigned to the correct gender label according to our manually created baseline. Recall measures how many of the contributors were correctly identified. F-score takes the harmonic average of these two metrics

Figure S2.

**Point estimates of distinctiveness in four model specifications.** Point estimates of distinctiveness with 95% CI for gender diversity, four variables of inclusion, and their interactions with gender diversity. Markers are numbered according to OLS models; coefficients are for one SD change in distinctiveness because of one SD change in independent variables. Panel a) shows the point estimates of different inclusion models based on the baseline game-level OLS models shown in the manuscript. Panel b) shows estimates for game-level OLS models with Random Effects for firms to estimate the effect of game specific characteristics and Panel c) with Fixed-Effects for firms to account for firm-level specific effects. Point estimates from models visualized in Panel d) are coming from firm-level aggregated data, where each variable is the average of all games produced by a given firm.

Figure S3.


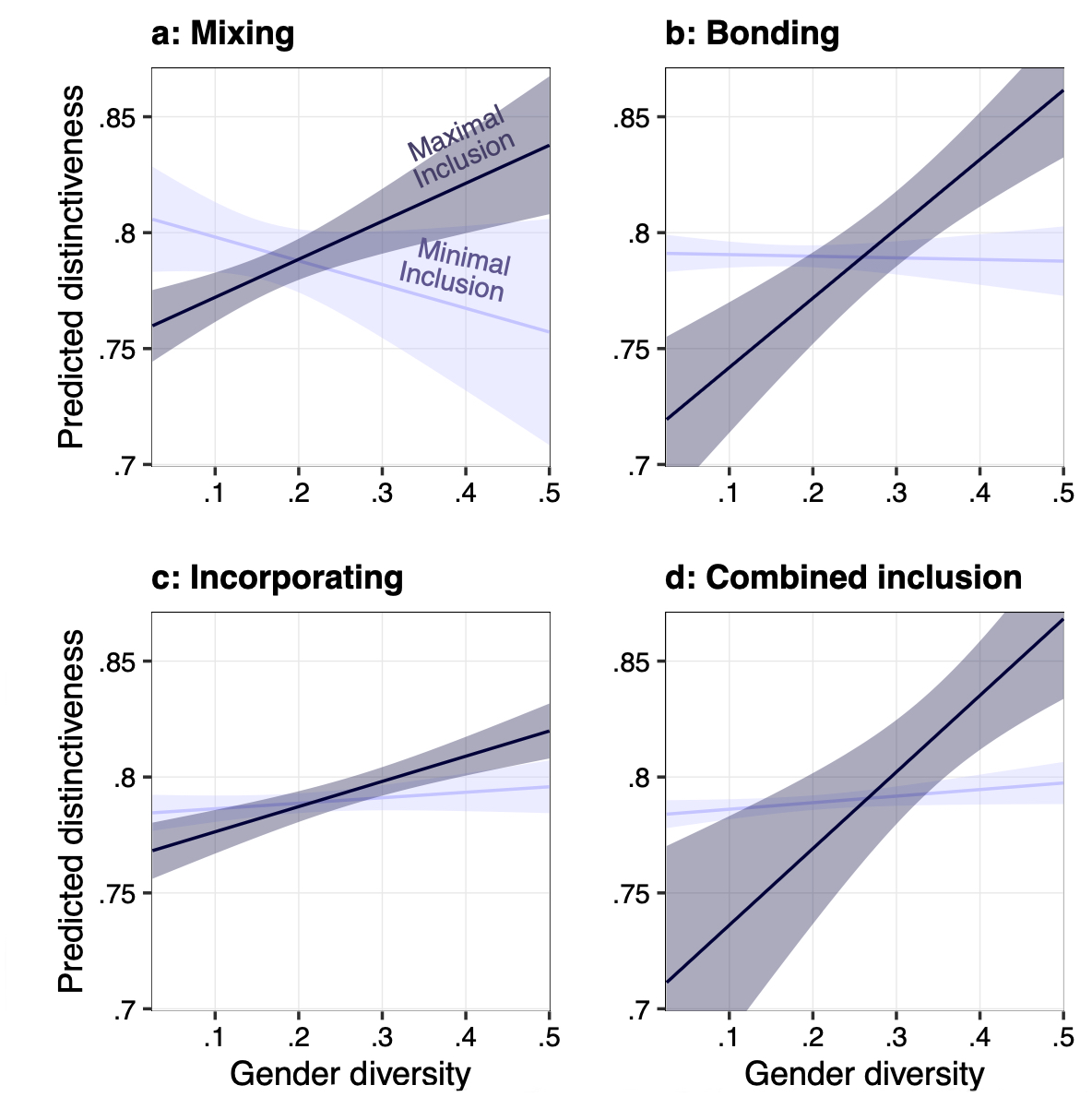


**Marginal predictions for all four inclusion variables** Game level OLS models’ predicted distinctiveness by by gender diversity at minimal and maximal levels of mixing inclusion with 95% CI, keeping all controls at their means.

Figure S4.

**Distribution of gender swapped diversity and inclusion metrics.** To assess the bias that name-based gender inferring method can introduce to our independent variables we randomly re-labelled 25 and 50 percent of unknown gendered team members to male in each game, and calculated Gender Diversity, Mixing, Bonding, Incorporating and Combined Inclusion. We repeated this process 100 times for each game and calculated the average of the resulting diversity and inclusion metrics. Blue histogram shows the original distribution, orange distribution is based on 25 percent re-labelled data, and green is based on 50 percent. Distributions indicate that Mixing and Incorporating are more sensitive to re-labelling than Bonding and the Combined Index.

Figure S5.

**Point estimates of distinctiveness based on OLS models ran on relabelled gender data.** To assess the impact of unknowns in project teams, we re-calculated our diversity and inclusion metrics 100 times by randomly relabelling 25 and 50 of unknown gendered team members to male. Blue indicates original point estimates of distinctiveness with 95% CI for gender diversity, yellow 25 percent of unknowns relabelled to male, and green 50.

Figure S6.


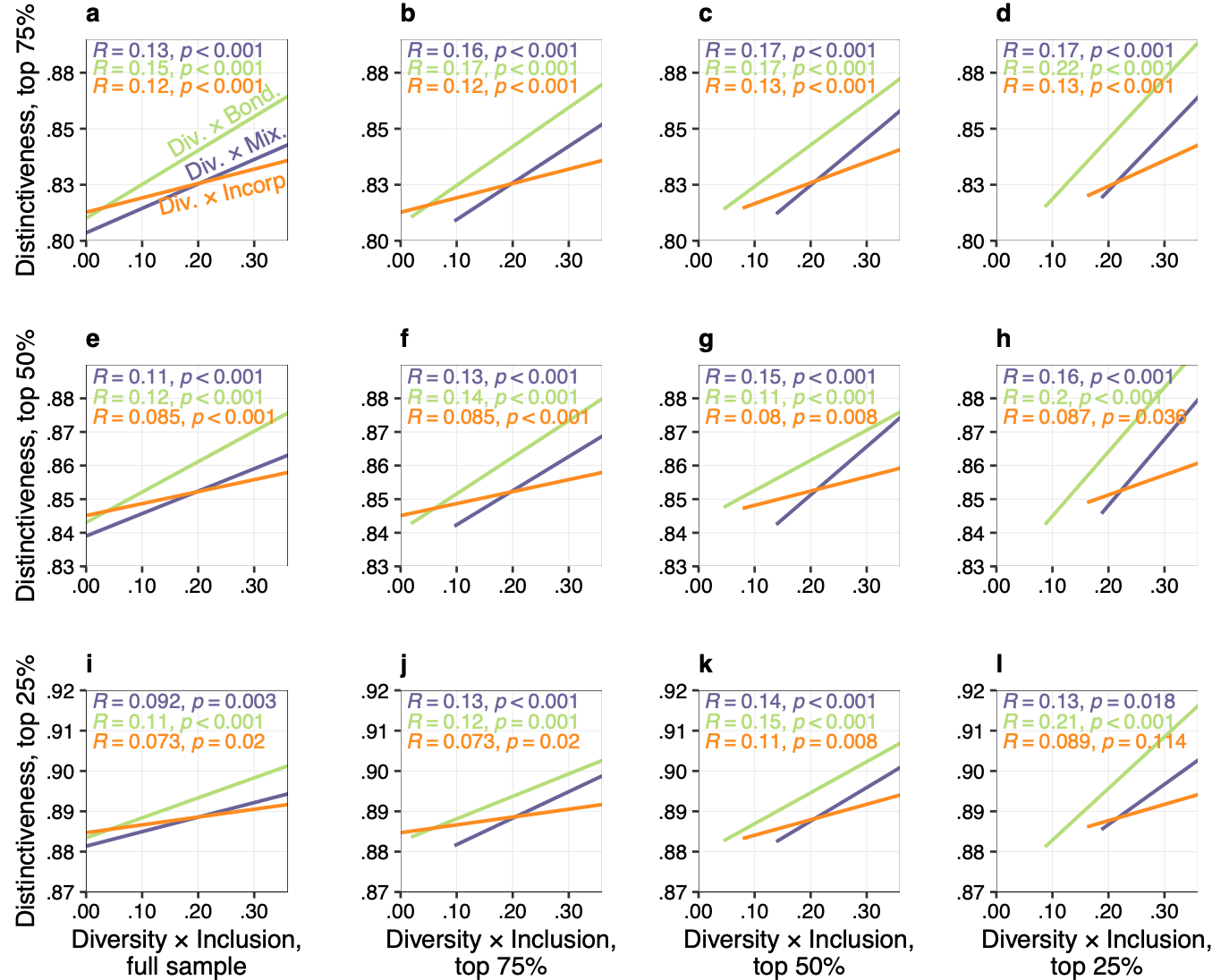


**Distinctiveness as a function of the interaction of diversity and inclusion.** Panels a though l show linear fits with varying restriction to the range of the product of Diversity and Inclusion metrics (top 75%, 50%, 25% of observations; marked on the x axis) and restriction to the range of Distinctiveness (top 75%, 50%, 25% of observations; marked on the y axis). Three inclusion metrics are marked by color; Pearson correlations and p values are shown separately by inclusion metric.

Figure S7.

**Prediction of Distinctiveness based on the interaction of Inclusion** (Mixing, Bonding, Incorporating and Combined Inclusion) **and Gender Diversity on different subsets of the database.** The figure shows model predictions for synthetic data, where we can compare the predicted distinctiveness of synthetic game projects as we manipulate diversity, and keep inclusion either at the minimum (0) or at the maximum (1). The database is divided by genre category (first row), production company size (second row) and published year (third row). Genres are split into “Action, adventure games” and “Simulation, sport, and strategy”. Production company size is defined by the number of games a production firm has published within our database. First group includes games that was published by the Top 250 most publishing companies, second group includes games belonging to the Bottom 250. Table shows key variables and main controls (except year and platform dummies). Games are grouped by published year as the following published between 1994-2001 or between 2001-2009.

Figure S8.


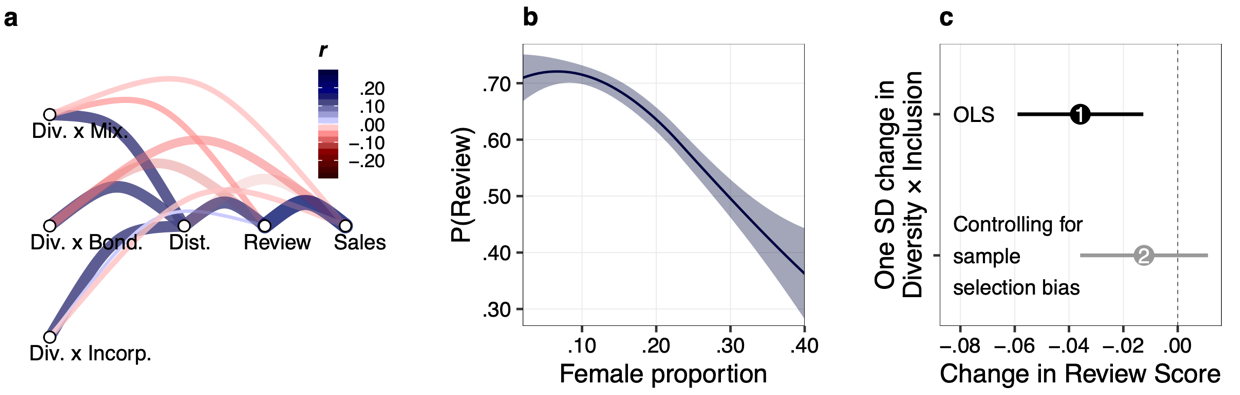


**Diversity, inclusion, and forms of success.** Panel a: Correlations among Diversity × Inclusion measures and game level outcomes. Edge color and thickness represents Pearson correlation as indicated by legend. Panel b: The probability of getting a review for the game as a function of the female proportion of developers in the team. Panel c: Point estimates of Review Score with 95% CI in an OLS model (marker 1; without controlling for selection bias) and a Heckman outcome model (marker 2; with controlling for selection bias). Coefficients represent one SD change in Review Score as a result of one SD change in Diversity × Combined inclusion. Non-significant estimates at p < .05 are shown in gray.

**Table S1. Descriptive Statistics of original data collected from mobygames.com** We collected data from the video game industry, relying on MobyGames.com. Our dataset contains 8,617 unique video games, with a list of each game’s developer teams, critic’s reviews, and stylistic elements such as genres, perspective (e.g., first-person shooter, role-playing) and the platforms it can be played on (e.g., PlayStation, Nintendo Switch, etc.). We also record each game’s developer studio, publishing house, and the year of the first release.

| Number of games | 8,617 |
| --- | --- |
| Years | 1993-2009 |
| Number of developers | 630,420 |
| Women | 119,826 (19%) |
| Men | 397,520 (63%) |
| Unknowns | 113,074 (18%) |

**Table S2. Applied filtering criteria on our game dataset.** For our analysis we only considered games which were published between 1993 and 2009, and had less than 2000 connection among team members, had at least one female team member, and less than 50% of team members gender could have been inferred. We excluded all re-released and mobile games. Since gender diversity is a key interest of our study, we had to exclude all those video games from our analysis which did not list team members’ full name and used only initials instead of first names. Our resulting database contains 4,011 video games.

| **Filtering Criteria** | **N** |
| --- | --- |
| All games | 8,617 |
| Published between 1993 and 2009 | 7,931 |
| 1<number of edges in the network <2000 | 4,771 |
| Ratio of unknowns in team < 0.5 | 4,654 |
| Number of women in team network >=1 | **4,011** |

**Table S3. Ratio of matching gender categories by Our method and Gender-Guesser.** Female contributors matched in 89.5%, and male contributors 96% of our sample.

| **Gender Guesser** | **Female** | **Male** | **Unknown** |
| --- | --- | --- | --- |
| **Our Method** |  |  |  |
| **Female** | 0.895 | 0.04 | 0.065 |
| **Male** | 0.005 | 0.96 | 0.035 |
| **Unknown** | 0.135 | 0.29 | 0.575 |

Table S4. Predicting Creativity with Mixing. Table shows key variables and main controls (except year and platform dummies). Model 1) Baseline Model with standardized variables Model 2) Developer firms Random-Effects 3) Developer firms with Fixed-effects (as dummies) 4) Developer firm level aggregated models

|  | | | | |
| --- | --- | --- | --- | --- |
|  | Distinctiveness | | | |
|  |  | | | |
|  | *OLS Models* | | | |
|  | Std rob SE | Dev firm RE | Dev firm FE dummies | Dev firm level std |
|  | (1) | (2) | (3) | (4) |
|  | | | | |
| Diversity | -0.143 | -0.122 | 0.021 | -0.361* |
|  | (0.110) | (0.106) | (0.118) | (0.192) |
|  |  |  |  |  |
| Mixing | -0.067** | -0.057** | -0.016 | -0.142** |
|  | (0.029) | (0.027) | (0.028) | (0.056) |
|  |  |  |  |  |
| Diversity:Mixing | 0.039** | 0.033* | 0.003 | 0.081*** |
|  | (0.018) | (0.018) | (0.020) | (0.031) |
| Team Size | -0.001*** | -0.001*** | -0.001*** | -0.001** |
|  | (0.0002) | (0.0002) | (0.0003) | (0.0004) |
|  |  |  |  |  |
| Newbies ratio | 0.141 | 0.131 | 0.159 | 0.045 |
|  | (0.116) | (0.113) | (0.133) | (0.241) |
|  |  |  |  |  |
| Games Tenure | 0.036 | 0.008 | 0.010 | 0.017 |
|  | (0.023) | (0.024) | (0.033) | (0.044) |
|  |  |  |  |  |
| Star developer | 0.108 | 0.284 | 0.324 | -0.377 |
|  | (0.311) | (0.386) | (0.310) | (0.243) |
|  |  |  |  |  |
| Single-firm production | 0.052 | 0.006 | -0.006 | -0.019 |
|  | (0.034) | (0.038) | (0.052) | (0.068) |
|  |  |  |  |  |
| Ratio of core | 0.220** | 0.187** | 0.115 | 0.449** |
|  | (0.087) | (0.082) | (0.096) | (0.211) |
|  |  |  |  |  |
| Number of countries | -0.033*** | -0.028*** | -0.024*** | -0.043*** |
|  | (0.006) | (0.006) | (0.007) | (0.014) |
|  |  |  |  |  |
|  |  |  |  |  |
|  | | | | |
| Observations | 4,011 | 4,011 | 3,368 | 1,354 |
| R2 | 0.110 | 0.224 | 0.474 | 0.163 |
| Adjusted R2 | 0.104 | 0.218 | 0.325 | 0.143 |
|  | | | | |
| *Note:* | *p**p***p<0.01 | | | |

**Table S5. Predicting Creativity as *Distinctivness* within various time ranges, 1,3,5,7 years with *Mixing*.** Table shows key variables and main controls (except year and platform dummies). Model shows standardized variables and Robust SE.

|  | | | | |
| --- | --- | --- | --- | --- |
|  | Distinctiveness | | | |
|  |  | | | |
|  | 1 year | 3 years | 5 years | 7 years |
|  | 1yr rob SE | 3yr rob SE | 5yr rob SE | 7yr rob SE |
|  | (1) | (2) | (3) | (4) |
|  | | | | |
| Diversity | -0.107 | -0.093 | -0.076 | -0.068 |
|  | (0.071) | (0.071) | (0.070) | (0.069) |
|  |  |  |  |  |
| Mixing | -0.055^***^ | -0.049^**^ | -0.043^**^ | -0.040^**^ |
|  | (0.021) | (0.021) | (0.020) | (0.020) |
| Diversity:Mixing | 0.266^**^ | 0.242^**^ | 0.212^*^ | 0.198^*^ |
|  | (0.114) | (0.113) | (0.112) | (0.111) |
|  |  |  |  |  |
| Team Size | -0.0001^***^ | -0.0001^***^ | -0.0001^***^ | -0.0001^***^ |
|  | (0.00001) | (0.00001) | (0.00001) | (0.00001) |
|  |  |  |  |  |
| Newbies ratio | 0.005 | 0.007 | 0.006 | 0.005 |
|  | (0.008) | (0.008) | (0.007) | (0.007) |
|  |  |  |  |  |
| Games Tenure | 0.002 | 0.002 | 0.002 | 0.002 |
|  | (0.002) | (0.001) | (0.001) | (0.001) |
|  |  |  |  |  |
| Star developer | 0.004 | 0.003 | 0.002 | 0.001 |
|  | (0.020) | (0.020) | (0.020) | (0.020) |
|  |  |  |  |  |
| Single-firm production | 0.002 | 0.002 | 0.003 | 0.003 |
|  | (0.002) | (0.002) | (0.002) | (0.002) |
|  |  |  |  |  |
| Ratio of core | 0.013^**^ | 0.013^**^ | 0.013^**^ | 0.013^**^ |
|  | (0.006) | (0.006) | (0.006) | (0.006) |
|  |  |  |  |  |
| Number of countries | -0.002^***^ | -0.002^***^ | -0.002^***^ | -0.002^***^ |
|  | (0.0004) | (0.0004) | (0.0004) | (0.0004) |
|  |  |  |  |  |
|  |  |  |  |  |
|  | | | | |
| Observations | 4,011 | 4,011 | 4,011 | 4,011 |
| R^2^ | 0.112 | 0.111 | 0.106 | 0.098 |
| Adjusted R^2^ | 0.105 | 0.104 | 0.099 | 0.091 |
|  | | | | |
| Note: | ^*^p^**^p^***^p<0.01 | | | |

**Table S6. Predicting *Creativity* with *Mixing* by genre category groups**. First group includes Action, adventure games, second Simulation, sport, and strategy. Table shows key variables and main controls (except year and platform dummies).

|  | Distinctiveness | |
| --- | --- | --- |
|  |  | |
|  |  | |
|  | Action, adventure | Simulation, sports, strategy |
|  | (1) | (2) |
|  | | |
| Diversity | -0.133^*^ | -0.010 |
|  | (0.074) | (0.089) |
|  |  |  |
| Mixing | -0.075^***^ | -0.025 |
|  | (0.022) | (0.026) |
|  |  |  |
| Team Size | -0.00002 | -0.0001^***^ |
|  | (0.00001) | (0.00002) |
|  |  |  |
| Newbies ratio | -0.006 | 0.021^**^ |
|  | (0.008) | (0.010) |
|  |  |  |
| Games Tenure | -0.004^***^ | 0.007^***^ |
|  | (0.002) | (0.002) |
|  |  |  |
| Star developer | 0.021 | -0.021 |
|  | (0.022) | (0.027) |
|  |  |  |
| Single-firm production | 0.002 | 0.003 |
|  | (0.002) | (0.003) |
|  |  |  |
| Ratio of core | 0.015^**^ | 0.005 |
|  | (0.007) | (0.008) |
|  |  |  |
| Number of countries | -0.001^***^ | -0.002^***^ |
|  | (0.0005) | (0.001) |
|  |  |  |
| Diversity:Mixing | 0.349^***^ | 0.145 |
|  | (0.118) | (0.141) |
|  |  |  |
|  | | |
| Observations | 2,381 | 2,371 |
| R^2^ | 0.150 | 0.132 |
| Adjusted R^2^ | 0.139 | 0.120 |
|  | | |
| Note: | ^*^p^**^p^***^p<0.01 | |

**Table S7. Predicting *Creativity* with *Mixing* by production company size.** Production company size is defined by the number of games a production firm has published within our database. First group includes games that was published by the Top 250 most publishing companies, second group includes games belonging to the Bottom 250. Table shows key variables and main controls (except year and platform dummies).

|  | | |
| --- | --- | --- |
|  | Distinctiveness | |
|  |  | |
|  | Top 1-250 | Top 251-smallest |
|  | (1) | (2) |
|  | | |
| Diversity | -0.060 | -0.136 |
|  | (0.110) | (0.093) |
|  |  |  |
| Mixing | -0.047 | -0.060^**^ |
|  | (0.033) | (0.028) |
|  |  |  |
| Team Size | -0.0001^***^ | -0.0001^***^ |
|  | (0.00002) | (0.00002) |
|  |  |  |
| Newbies ratio | 0.005 | 0.016 |
|  | (0.012) | (0.011) |
|  |  |  |
| Games Tenure | 0.004^*^ | 0.001 |
|  | (0.002) | (0.002) |
|  |  |  |
| Star developer | 0.028 | -0.068 |
|  | (0.026) | (0.047) |
|  |  |  |
| Single-firm production | 0.008^**^ | -0.003 |
|  | (0.003) | (0.004) |
|  |  |  |
| Ratio of core | 0.008 | 0.026^***^ |
|  | (0.008) | (0.010) |
|  |  |  |
| Number of countries | -0.003^***^ | -0.002^**^ |
|  | (0.001) | (0.001) |
|  |  |  |
| Diversity:Mixing | 0.186 | 0.337^**^ |
|  | (0.175) | (0.148) |
|  |  |  |
|  | | |
| Observations | 2,186 | 1,825 |
| R^2^ | 0.107 | 0.132 |
| Adjusted R^2^ | 0.094 | 0.117 |
|  | | |
| Note: | ^*^p^**^p^***^p<0.01 | |

**Table S8. Predicting *Creativity* with *Mixing* by production year category groups**. First group includes games published between 1994-2001 second group includes 2001-2009. Table shows key variables and main controls (except year and platform dummies).

|  | Distinctiveness | |
| --- | --- | --- |
|  |  | |
|  | 1994-2001 | 2002-2009 |
|  | (1) | (2) |
|  | | |
| Diversity | -0.156 | -0.037 |
|  | (0.099) | (0.102) |
|  |  |  |
| Mixing | -0.064^**^ | -0.044 |
|  | (0.028) | (0.032) |
|  |  |  |
| Team Size | -0.00004^**^ | -0.0001^***^ |
|  | (0.00002) | (0.00002) |
|  |  |  |
| Newbies ratio | 0.002 | 0.023^*^ |
|  | (0.011) | (0.012) |
|  |  |  |
| Games Tenure | 0.004 | 0.003 |
|  | (0.003) | (0.002) |
|  |  |  |
| Star developer |  | 0.010 |
|  |  | (0.023) |
|  |  |  |
| Single-firm production | 0.004 | 0.001 |
|  | (0.003) | (0.004) |
|  |  |  |
| Ratio of core | 0.007 | 0.023^***^ |
|  | (0.009) | (0.008) |
|  |  |  |
| Number of countries | -0.004^***^ | -0.002^***^ |
|  | (0.001) | (0.001) |
|  |  |  |
| Diversity:Mixing | 0.392^**^ | 0.116 |
|  | (0.156) | (0.164) |
|  |  |  |
|  | | |
| Observations | 2,137 | 1,874 |
| R^2^ | 0.079 | 0.123 |
| Adjusted R^2^ | 0.070 | 0.113 |
|  | | |
| Note: | ^*^p^**^p^***^p<0.01 | |

**Table S9. Predicting *Novelty* as the log number of newly introduced style-pairings and the ratio of newly introduced style-pairings from all style-pairings based on Hofstra et al, 2020, Yang et al 2022 with *Mixing***. Table shows key variables and main controls (except year and platform dummies). Model 1) Baseline Model with standardized variables and Robust SE Model 2) Baseline Model with standardized variables and Clustered SE.

|  | Novelty | | | |
| --- | --- | --- | --- | --- |
|  |  | | | |
|  | log10(Number of new pairs + 1) | | Ratio of new pairs | |
|  | rob SE | clus SE | rob SE | clus SE |
|  | (1) | (2) | (3) | (4) |
|  | | | | |
| Diversity | -0.565 | -0.565 | -0.368 | -0.368 |
|  | (0.409) | (0.409) | (0.262) | (0.262) |
|  |  |  |  |  |
| Mixing | -0.257^**^ | -0.257^**^ | -0.226^***^ | -0.226^***^ |
|  | (0.124) | (0.124) | (0.081) | (0.081) |
|  |  |  |  |  |
| Diversity:Mixing | 1.197^*^ | 1.197^*^ | 0.840^**^ | 0.840^**^ |
|  | (0.654) | (0.654) | (0.418) | (0.418) |
| Team Size | -0.00002 | -0.00002 | -0.0002^***^ | -0.0002^***^ |
|  | (0.0001) | (0.0001) | (0.00004) | (0.00004) |
|  |  |  |  |  |
| Newbies ratio | -0.022 | -0.022 | -0.020 | -0.020 |
|  | (0.041) | (0.041) | (0.027) | (0.027) |
|  |  |  |  |  |
| Games Tenure | -0.013^*^ | -0.013^*^ | -0.010^**^ | -0.010^**^ |
|  | (0.008) | (0.008) | (0.005) | (0.005) |
|  |  |  |  |  |
| Star developer | 0.068 | 0.068 | 0.020 | 0.020 |
|  | (0.153) | (0.153) | (0.058) | (0.058) |
|  |  |  |  |  |
| Single-firm production | 0.034^***^ | 0.034^***^ | 0.024^***^ | 0.024^***^ |
|  | (0.012) | (0.012) | (0.008) | (0.008) |
|  |  |  |  |  |
| Ratio of core | -0.014 | -0.014 | -0.014 | -0.014 |
|  | (0.030) | (0.030) | (0.020) | (0.020) |
|  |  |  |  |  |
| Number of countries | -0.003 | -0.003 | -0.004^***^ | -0.004^***^ |
|  | (0.002) | (0.002) | (0.001) | (0.001) |
|  |  |  |  |  |
|  |  |  |  |  |
|  | | | | |
| Observations | 4,011 | 4,011 | 4,001 | 4,001 |
| R^2^ | 0.081 | 0.081 | 0.110 | 0.110 |
| Adjusted R^2^ | 0.074 | 0.074 | 0.103 | 0.103 |
|  | | | | |
| Note: | ^*^p^**^p^***^p<0.01 | | | |

**Table S10. Predicting *Creativity* with *Bonding*.** Table shows key variables and main controls (except year and platform dummies). Model 1) Baseline Model with standardized variables Model 2) Developer firms Random-Effects 3) Developer firms with Fixed-effects (as dummies) 4) Developer firm level aggregated models

|  | Distinctiveness | | | |
| --- | --- | --- | --- | --- |
|  | *OLS Models* | | | |
|  | Std rob SE | Dev firm RE | Dev firm FE dummies | Dev firm level std |
|  | (1) | (2) | (3) | (4) |
| Diversity | -0.010 | -0.004 | 0.049 | -0.092 |
|  | (0.032) | (0.031) | (0.039) | (0.058) |
| Bonding | -0.134^***^ | -0.134^***^ | -0.103^**^ | -0.172^**^ |
|  | (0.040) | (0.041) | (0.048) | (0.071) |
| Diversity: Bonding | 0.057^***^ | 0.051^***^ | 0.018 | 0.094^***^ |
|  | (0.015) | (0.015) | (0.017) | (0.024) |
| Team Size | -0.001^***^ | -0.001^***^ | -0.001^***^ | -0.001^**^ |
|  | (0.0002) | (0.0002) | (0.0003) | (0.0004) |
| Newbies ratio | 0.169 | 0.154 | 0.176 | 0.087 |
|  | (0.115) | (0.113) | (0.133) | (0.237) |
| Games tenure | 0.038^*^ | 0.009 | 0.012 | 0.027 |
|  | (0.023) | (0.024) | (0.033) | (0.043) |
| Star developer | 0.146 | 0.307 | 0.327 | -0.327 |
|  | (0.307) | (0.386) | (0.305) | (0.229) |
| Single-firm production | 0.050 | 0.005 | -0.005 | -0.029 |
|  | (0.034) | (0.038) | (0.052) | (0.068) |
| Ratio of core | 0.216^**^ | 0.184^**^ | 0.113 | 0.457^**^ |
|  | (0.087) | (0.082) | (0.096) | (0.206) |
| Number of countries | -0.032^***^ | -0.028^***^ | -0.024^***^ | -0.038^***^ |
|  | (0.006) | (0.006) | (0.007) | (0.014) |
| N | 4,011 | 4,011 | 3,368 | 1,354 |
| R^2^ | 0.113 | 0.225 | 0.475 | 0.170 |
| Adjusted R^2^ | 0.106 | 0.219 | 0.327 | 0.151 |
| *Note:* | ^*^p<0.1; ^**^p<0.05; ^***^p<0.01 | | | |

**Table S11. Predicting Creativity as *Distinctivness* within various time ranges, 1,3,5,7 years with *Bonding*.** Table shows key variables and main controls (except year and platform dummies). Model shows standardized variables and Robust SE.

|  | | | | |
| --- | --- | --- | --- | --- |
|  | Distinctiveness | | | |
|  |  | | | |
|  | 1 year | 3 years | 5 years | 7 years |
|  | 1yr rob SE | 3yr rob SE | 5yr rob SE | 7yr rob SE |
|  | (1) | (2) | (3) | (4) |
|  | | | | |
| Diversity | -0.008 | -0.006 | -0.006 | -0.008 |
|  | (0.022) | (0.021) | (0.021) | (0.021) |
|  |  |  |  |  |
| Bonding | -0.078^***^ | -0.073^***^ | -0.070^***^ | -0.068^***^ |
|  | (0.022) | (0.021) | (0.021) | (0.021) |
|  |  |  |  |  |
| Diversity:Bonding | 0.294^***^ | 0.280^***^ | 0.273^***^ | 0.270^***^ |
|  | (0.075) | (0.073) | (0.072) | (0.072) |
| Team Size | -0.0001^***^ | -0.0001^***^ | -0.0001^***^ | -0.0001^***^ |
|  | (0.00001) | (0.00001) | (0.00001) | (0.00001) |
|  |  |  |  |  |
| Newbies ratio | 0.007 | 0.008 | 0.008 | 0.007 |
|  | (0.008) | (0.007) | (0.007) | (0.007) |
|  |  |  |  |  |
| Games Tenure | 0.002 | 0.002 | 0.002 | 0.002 |
|  | (0.002) | (0.001) | (0.001) | (0.001) |
|  |  |  |  |  |
| Star developer | 0.007 | 0.005 | 0.004 | 0.003 |
|  | (0.020) | (0.020) | (0.020) | (0.020) |
|  |  |  |  |  |
| Single-firm production | 0.002 | 0.002 | 0.003 | 0.003 |
|  | (0.002) | (0.002) | (0.002) | (0.002) |
|  |  |  |  |  |
| Ratio of core | 0.013^**^ | 0.013^**^ | 0.013^**^ | 0.012^**^ |
|  | (0.006) | (0.006) | (0.006) | (0.006) |
|  |  |  |  |  |
| Number of countries | -0.002^***^ | -0.002^***^ | -0.002^***^ | -0.002^***^ |
|  | (0.0004) | (0.0004) | (0.0004) | (0.0004) |
|  |  |  |  |  |
|  |  |  |  |  |
|  | | | | |
| Observations | 4,011 | 4,011 | 4,011 | 4,011 |
| R^2^ | 0.114 | 0.113 | 0.108 | 0.101 |
| Adjusted R^2^ | 0.107 | 0.107 | 0.101 | 0.094 |
|  | | | | |
| Note: | ^*^p^**^p^***^p<0.01 | | | |

**Table S12. Predicting *Creativity* with *Bonding* by genre category groups.** First group includes Action, adventure games, second Simulation, sport, and strategy. Table shows key variables and main controls (except year and platform dummies).

|  | | |
| --- | --- | --- |
|  | Distinctiveness | |
|  |  | |
|  |  | |
|  | Action, adventure | Simulation, sports, strategy |
|  | (1) | (2) |
|  | | |
| Diversity | 0.052^**^ | 0.015 |
|  | (0.023) | (0.029) |
|  |  |  |
| Bonding | -0.093^***^ | -0.070^**^ |
|  | (0.023) | (0.028) |
|  |  |  |
| Diversity:Bonding | 0.247^***^ | 0.277^***^ |
|  | (0.079) | (0.092) |
|  |  |  |
|  | (0.00001) | (0.00002) |
|  |  |  |
| Newbies ratio | -0.003 | 0.023^**^ |
|  | (0.008) | (0.010) |
|  |  |  |
| Games Tenure | -0.004^***^ | 0.007^***^ |
|  | (0.002) | (0.002) |
|  |  |  |
| Star developer | 0.021 | -0.019 |
|  | (0.022) | (0.027) |
|  |  |  |
| Single-firm production | 0.002 | 0.003 |
|  | (0.002) | (0.003) |
|  |  |  |
| Ratio of core | 0.014^**^ | 0.005 |
|  | (0.007) | (0.008) |
|  |  |  |
| Number of countries | -0.001^***^ | -0.002^***^ |
|  | (0.0005) | (0.001) |
| Observations | 2,381 | 2,371 |
| R^2^ | 0.152 | 0.135 |
| Adjusted R^2^ | 0.140 | 0.123 |
|  | | |
| Note: | ^*^p^**^p^***^p<0.01 | |

**Table S13. Predicting *Creativity* with *Bonding* by production company size**. Production company size is defined by the number of games a production firm has published within our database. First group includes games that was published by the Top 250 most publishing companies, second group includes games belonging to the Bottom 250. Table shows key variables and main controls (except year and platform dummies).

|  | | |
| --- | --- | --- |
|  | Distinctiveness | |
|  |  | |
|  | Top 1-250 | Top 251-smallest |
|  | (1) | (2) |
|  | | |
| Diversity | 0.016 | -0.024 |
|  | (0.031) | (0.032) |
|  |  |  |
| Bonding | -0.107^***^ | -0.053^*^ |
|  | (0.032) | (0.032) |
|  |  |  |
| Diversity:Bonding | 0.299^***^ | 0.307^***^ |
|  | (0.106) | (0.106) |
| Team Size | -0.0001^***^ | -0.0001^***^ |
|  | (0.00002) | (0.00002) |
|  |  |  |
| Newbies ratio | 0.007 | 0.018^*^ |
|  | (0.012) | (0.011) |
|  |  |  |
| Games Tenure | 0.004^*^ | 0.002 |
|  | (0.002) | (0.002) |
|  |  |  |
| Star developer | 0.028 | -0.063 |
|  | (0.026) | (0.047) |
|  |  |  |
| Single-firm production | 0.007^**^ | -0.004 |
|  | (0.003) | (0.004) |
|  |  |  |
| Ratio of core | 0.007 | 0.025^**^ |
|  | (0.008) | (0.010) |
|  |  |  |
| Number of countries | -0.003^***^ | -0.002^**^ |
|  | (0.001) | (0.001) |
|  |  |  |
|  |  |  |
|  | | |
| Observations | 2,186 | 1,825 |
| R^2^ | 0.111 | 0.135 |
| Adjusted R^2^ | 0.098 | 0.120 |
|  | | |
| Note: | ^*^p^**^p^***^p<0.01 | |

**Table S14. Predicting *Creativity* with *Bonding* by production year category groups.** First group includes games published between 1994-2001 second group includes 2001-2009. Table shows key variables and main controls (except year and platform dummies).

|  | | |
| --- | --- | --- |
|  | Distinctiveness | |
|  |  | |
|  | 1994-2001 | 2002-2009 |
|  | (1) | (2) |
|  | | |
| Diversity | -0.034 | 0.025 |
|  | (0.030) | (0.033) |
|  |  |  |
| Bonding | -0.098^***^ | -0.055 |
|  | (0.030) | (0.034) |
|  |  |  |
| Diversity:Bonding | 0.459^***^ | 0.122 |
|  | (0.100) | (0.112) |
| Team Size | -0.00004^**^ | -0.0001^***^ |
|  | (0.00002) | (0.00002) |
|  |  |  |
| Newbies ratio | 0.006 | 0.023^*^ |
|  | (0.011) | (0.012) |
|  |  |  |
| Games Tenure | 0.004 | 0.003 |
|  | (0.003) | (0.002) |
|  |  |  |
| Star developer |  | 0.009 |
|  |  | (0.023) |
|  |  |  |
| Single-firm production | 0.005 | 0.001 |
|  | (0.003) | (0.004) |
|  |  |  |
| Ratio of core | 0.006 | 0.023^***^ |
|  | (0.009) | (0.008) |
|  |  |  |
| Number of countries | -0.004^***^ | -0.002^***^ |
|  | (0.001) | (0.001) |
|  |  |  |
|  |  |  |
|  | | |
| Observations | 2,137 | 1,874 |
| R^2^ | 0.086 | 0.123 |
| Adjusted R^2^ | 0.077 | 0.113 |
|  | | |
| Note: | ^*^p^**^p^***^p<0.01 | |

**Table S15. Predicting *Novelty* as the log number of newly introduced style-pairings and the ratio of newly introduced style-pairings from all style-pairings based on Hofstra et al, 2020, Yang et al 2022 with *Bonding*.** Table shows key variables and main controls (except year and platform dummies). Model 1) Baseline Model with standardized variables and Robust SE Model 2) Baseline Model with standardized variables and Clustered SE.

|  | | | | |
| --- | --- | --- | --- | --- |
|  | Novelty | | | |
|  |  | | | |
|  | log10(Number of new pairs + 1) | | Ratio of new pairs | |
|  | rob SE | clus SE | rob SE | clus SE |
|  | (1) | (2) | (3) | (4) |
|  | | | | |
| Diversity | 0.029 | 0.029 | -0.033 | -0.033 |
|  | (0.115) | (0.115) | (0.115) | (0.075) |
|  |  |  |  |  |
| Bonding | -0.252^**^ | -0.252^**^ | -0.298^**^ | -0.298^***^ |
|  | (0.120) | (0.120) | (0.120) | (0.078) |
|  |  |  |  |  |
| Diversity:Bonding | 0.802^**^ | 0.802^**^ | 0.968^**^ | 0.968^***^ |
|  | (0.401) | (0.401) | (0.401) | (0.271) |
| Team Size | -0.00001 | -0.00001 | -0.0002^**^ | -0.0002^***^ |
|  | (0.0001) | (0.0001) | (0.0001) | (0.00004) |
|  |  |  |  |  |
| Newbies ratio | -0.014 | -0.014 | -0.015 | -0.015 |
|  | (0.041) | (0.041) | (0.041) | (0.027) |
|  |  |  |  |  |
| Games Tenure | -0.013^*^ | -0.013^*^ | -0.010 | -0.010^**^ |
|  | (0.008) | (0.008) | (0.008) | (0.005) |
|  |  |  |  |  |
| Star developer | 0.072 | 0.072 | 0.025 | 0.025 |
|  | (0.154) | (0.154) | (0.154) | (0.058) |
|  |  |  |  |  |
| Single-firm production | 0.033^***^ | 0.033^***^ | 0.023^*^ | 0.023^***^ |
|  | (0.012) | (0.012) | (0.012) | (0.008) |
|  |  |  |  |  |
| Ratio of core | -0.016 | -0.016 | -0.017 | -0.017 |
|  | (0.030) | (0.030) | (0.030) | (0.020) |
|  |  |  |  |  |
| Number of countries | -0.004 | -0.004 | -0.004^*^ | -0.004^***^ |
|  | (0.002) | (0.002) | (0.002) | (0.001) |
|  |  |  |  |  |
|  | | | | |
| Observations | 4,011 | 4,011 | 4,001 | 4,001 |
| R^2^ | 0.081 | 0.081 | 0.111 | 0.111 |
| Adjusted R^2^ | 0.073 | 0.073 | 0.104 | 0.104 |
|  | | | | |
| Note: | ^*^p^**^p^***^p<0.01 | | | |

**Table S16. Predicting *Creativity* with *Incorporating*.** Table shows key variables and main controls (except year and platform dummies). Model 1) Baseline Model with standardized variables Model 2) Developer firms Random-Effects 3) Developer firms with Fixed-effects (as dummies) 4) Developer firm level aggregated models.

|  | Distinctiveness | | | | |
| --- | --- | --- | --- | --- | --- |
|  | *OLS Models* | | | | |
|  | Std rob SE | Dev firm RE | Dev firm  FE dummies | | Dev firm level std |
|  | (1) | (2) | (3) | (4) | |
| Diversity | 0.033 | 0.027 | 0.012 | 0.060 | |
|  | (0.026) | (0.026) | (0.029) | (0.048) | |
| Incorporating | -0.073^*^ | -0.069^*^ | -0.052 | -0.091 | |
|  | (0.038) | (0.039) | (0.042) | (0.073) | |
| Diversity:  Incorporating | 0.038^**^ | 0.033^**^ | 0.020 | 0.043^*^ | |
|  | (0.015) | (0.016) | (0.016) | (0.025) | |
| Team Size | -0.001^***^ | -0.001^***^ | -0.001^***^ | -0.001^**^ | |
|  | (0.0002) | (0.0002) | (0.0003) | (0.0004) | |
| Newbies ratio | 0.160 | 0.146 | 0.158 | 0.094 | |
|  | (0.115) | (0.113) | (0.133) | (0.242) | |
| Games tenure | 0.037 | 0.008 | 0.009 | 0.019 | |
|  | (0.023) | (0.024) | (0.033) | (0.044) | |
| Star developer | 0.114 | 0.293 | 0.328 | -0.358 | |
|  | (0.312) | (0.381) | (0.309) | (0.245) | |
| Single-firm  production | 0.050 | 0.006 | -0.008 | -0.019 | |
|  | (0.034) | (0.038) | (0.052) | (0.069) | |
| Ratio of core | 0.186 | 0.182^*^ | 0.153 | 0.398 | |
|  | (0.122) | (0.109) | (0.135) | (0.291) | |
| Number of countries | -0.033^***^ | -0.029^***^ | -0.024^***^ | -0.042^***^ | |
|  | (0.006) | (0.006) | (0.007) | (0.014) | |
| N | 4,011 | 4,011 | 3,368 | 1,354 | |
| R^2^ | 0.111 | 0.224 | 0.474 | 0.159 | |
| Adjusted R^2^ | 0.104 | 0.218 | 0.326 | 0.139 | |
| *Note:* | ^*^p<0.1; ^**^p<0.05; ^***^p<0.01 | | | | |

**Table S17. Predicting Creativity as *Distinctivness* within various time ranges, 1,3,5,7 years with *Incorporating*.** Table shows key variables and main controls (except year and platform dummies). Model shows standardized variables and Robust SE.

|  | | | | |
| --- | --- | --- | --- | --- |
|  | Distinctiveness | | | |
|  |  | | | |
|  | 1 year | 3 years | 5 years | 7 years |
|  | 1yr rob SE | 3yr rob SE | 5yr rob SE | 7yr rob SE |
|  | (1) | (2) | (3) | (4) |
|  | | | | |
| Diversity | 0.018 | 0.021 | 0.021 | 0.021 |
|  | (0.017) | (0.017) | (0.017) | (0.017) |
|  |  |  |  |  |
| Incorporating | -0.019^**^ | -0.018^**^ | -0.018^**^ | -0.017^**^ |
|  | (0.009) | (0.008) | (0.008) | (0.008) |
|  |  |  |  |  |
| Diversity:Incorporating | 0.087^***^ | 0.079^**^ | 0.077^**^ | 0.075^**^ |
|  | (0.032) | (0.031) | (0.031) | (0.031) |
| Team Size | -0.0001^***^ | -0.0001^***^ | -0.0001^***^ | -0.0001^***^ |
|  | (0.00001) | (0.00001) | (0.00001) | (0.00001) |
|  |  |  |  |  |
| Newbies ratio | 0.007 | 0.008 | 0.007 | 0.006 |
|  | (0.008) | (0.007) | (0.007) | (0.007) |
|  |  |  |  |  |
| Games Tenure | 0.002 | 0.002 | 0.002 | 0.002 |
|  | (0.002) | (0.001) | (0.001) | (0.001) |
|  |  |  |  |  |
| Star developer | 0.005 | 0.003 | 0.002 | 0.001 |
|  | (0.020) | (0.020) | (0.020) | (0.020) |
|  |  |  |  |  |
| Single-firm production | 0.002 | 0.002 | 0.003 | 0.003 |
|  | (0.002) | (0.002) | (0.002) | (0.002) |
|  |  |  |  |  |
| Ratio of core | 0.011 | 0.011 | 0.012 | 0.011 |
|  | (0.008) | (0.008) | (0.008) | (0.008) |
|  |  |  |  |  |
| Number of countries | -0.002^***^ | -0.002^***^ | -0.002^***^ | -0.002^***^ |
|  | (0.0004) | (0.0004) | (0.0004) | (0.0004) |
|  |  |  |  |  |
|  |  |  |  |  |
|  | | | | |
| Observations | 4,011 | 4,011 | 4,011 | 4,011 |
| R^2^ | 0.112 | 0.111 | 0.106 | 0.098 |
| Adjusted R^2^ | 0.105 | 0.104 | 0.099 | 0.091 |
|  | | | | |
| Note: | ^*^p^**^p^***^p<0.01 | | | |

**Table S18. Predicting *Creativity* with *Incorporating* by genre category groups**. First group includes Action, adventure games, second Simulation, sport, and strategy. Table shows key variables and main controls (except year and platform dummies).

|  | | |
| --- | --- | --- |
|  | Distinctiveness | |
|  |  | |
|  | Action, adventure | Simulation, sports, strategy |
|  | (1) | (2) |
|  | | |
| Diversity | 0.047^**^ | 0.043^*^ |
|  | (0.019) | (0.024) |
|  |  |  |
| Incorporating | -0.012 | -0.024^**^ |
|  | (0.010) | (0.012) |
|  |  |  |
| Diversity:Incorporating | 0.070^**^ | 0.089^**^ |
|  | (0.034) | (0.043) |
| Team Size | -0.00002^*^ | -0.0001^***^ |
|  | (0.00001) | (0.00002) |
|  |  |  |
| Newbies ratio | -0.005 | 0.023^**^ |
|  | (0.008) | (0.010) |
|  |  |  |
| Games Tenure | -0.004^**^ | 0.007^***^ |
|  | (0.002) | (0.002) |
|  |  |  |
| Star developer | 0.021 | -0.020 |
|  | (0.022) | (0.027) |
|  |  |  |
| Single-firm production | 0.002 | 0.003 |
|  | (0.002) | (0.003) |
|  |  |  |
| Ratio of core | 0.007 | 0.009 |
|  | (0.009) | (0.011) |
|  |  |  |
| Number of countries | -0.001^***^ | -0.002^***^ |
|  | (0.0005) | (0.001) |
|  |  |  |
|  |  |  |
|  | | |
| Observations | 2,381 | 2,371 |
| R^2^ | 0.148 | 0.133 |
| Adjusted R^2^ | 0.136 | 0.121 |
|  | | |
| Note: | ^*^p^**^p^***^p<0.01 | |

**Table S19. Predicting *Creativity* with *Incorporating* by production company size.** Production company size is defined by the number of games a production firm has published within our database. First group includes games that was published by the Top 250 most publishing companies, second group includes games belonging to the Bottom 250. Table shows key variables and main controls (except year and platform dummies).

|  | | |
| --- | --- | --- |
|  | Distinctiveness | |
|  |  | |
|  | Top 1-250 | Top 251-smallest |
|  | (1) | (2) |
|  | | |
| Diversity | 0.028 | 0.024 |
|  | (0.026) | (0.026) |
|  |  |  |
| Incorporating | -0.013 | -0.024^*^ |
|  | (0.013) | (0.014) |
| Diversity:Incorporating | 0.058 | 0.110^**^ |
|  | (0.047) | (0.049) |
|  |  |  |
| Team Size | -0.0001^***^ | -0.0001^***^ |
|  | (0.00002) | (0.00002) |
|  |  |  |
| Newbies ratio | 0.005 | 0.018^*^ |
|  | (0.012) | (0.011) |
|  |  |  |
| Games Tenure | 0.004^*^ | 0.002 |
|  | (0.002) | (0.002) |
|  |  |  |
| Star developer | 0.028 | -0.066 |
|  | (0.026) | (0.047) |
|  |  |  |
| Single-firm production | 0.007^**^ | -0.003 |
|  | (0.003) | (0.004) |
|  |  |  |
| Ratio of core | 0.008 | 0.022 |
|  | (0.012) | (0.013) |
|  |  |  |
| Number of countries | -0.003^***^ | -0.002^**^ |
|  | (0.001) | (0.001) |
|  |  |  |
|  |  |  |
|  | | |
| Observations | 2,186 | 1,825 |
| R^2^ | 0.107 | 0.132 |
| Adjusted R^2^ | 0.094 | 0.117 |
|  | | |
| Note: | ^*^p^**^p^***^p<0.01 | |

**Table S20. Predicting *Creativity* with *Incorporating* by production year category groups.** First group includes games published between 1994-2001 second group includes 2001-2009. Table shows key variables and main controls (except year and platform dummies).

|  | | |
| --- | --- | --- |
|  | Distinctiveness | |
|  |  | |
|  | 1994-2001 | 2002-2009 |
|  | (1) | (2) |
|  | | |
| Diversity | 0.032 | 0.014 |
|  | (0.025) | (0.027) |
|  |  |  |
| Incorporating | -0.022^*^ | -0.015 |
|  | (0.012) | (0.015) |
| Diversity:Incorporating | 0.122^***^ | 0.046 |
|  | (0.045) | (0.050) |
|  |  |  |
| Team Size | -0.00005^**^ | -0.0001^***^ |
|  | (0.00002) | (0.00002) |
|  |  |  |
| Newbies ratio | 0.004 | 0.023^*^ |
|  | (0.011) | (0.012) |
|  |  |  |
| Games Tenure | 0.004 | 0.002 |
|  | (0.003) | (0.002) |
|  |  |  |
| Star developer |  | 0.010 |
|  |  | (0.023) |
|  |  |  |
| Single-firm production | 0.004 | 0.001 |
|  | (0.003) | (0.004) |
|  |  |  |
| Ratio of core | -0.002 | 0.028^**^ |
|  | (0.012) | (0.013) |
|  |  |  |
| Number of countries | -0.004^***^ | -0.002^***^ |
|  | (0.001) | (0.001) |
|  |  |  |
|  |  |  |
|  | | |
| Observations | 2,137 | 1,874 |
| R^2^ | 0.080 | 0.122 |
| Adjusted R^2^ | 0.070 | 0.112 |
|  | | |
| Note: | ^*^p^**^p^***^p<0.01 | |

**Table S21. Predicting *Novelty* as the log number of newly introduced style-pairings and the ratio of newly introduced style-pairings from all style-pairings based on Hofstra et al, 2020, Yang et al 2022 with *Incorporating*.** Table shows key variables and main controls (except year and platform dummies). Model 1) Baseline Model with standardized variables and Robust SE Model 2) Baseline Model with standardized variables and Clustered SE.

|  | | | | |
| --- | --- | --- | --- | --- |
|  | Novelty | | | |
|  |  | | | |
|  | log10(Number of new pairs + 1) | | Ratio of new pairs | |
|  | rob SE | clus SE | rob SE | clus SE |
|  | (1) | (2) | (3) | (4) |
|  | | | | |
| Diversity | 0.081 | 0.081 | 0.035 | 0.035 |
|  | (0.095) | (0.095) | (0.062) | (0.062) |
|  |  |  |  |  |
| Incorporating | 0.016 | 0.016 | -0.042 | -0.042 |
|  | (0.049) | (0.049) | (0.032) | (0.032) |
| Diversity:Incorporating | 0.131 | 0.131 | 0.225^*^ | 0.225^*^ |
|  | (0.183) | (0.183) | (0.116) | (0.116) |
|  |  |  |  |  |
| Team Size | -0.00004 | -0.00004 | -0.0002^***^ | -0.0002^***^ |
|  | (0.0001) | (0.0001) | (0.00004) | (0.00004) |
|  |  |  |  |  |
| Newbies ratio | -0.021 | -0.021 | -0.018 | -0.018 |
|  | (0.041) | (0.041) | (0.027) | (0.027) |
|  |  |  |  |  |
| Games Tenure | -0.012 | -0.012 | -0.010^**^ | -0.010^**^ |
|  | (0.008) | (0.008) | (0.005) | (0.005) |
|  |  |  |  |  |
| Star developer | 0.062 | 0.062 | 0.019 | 0.019 |
|  | (0.153) | (0.153) | (0.057) | (0.057) |
|  |  |  |  |  |
| Single-firm production | 0.033^***^ | 0.033^***^ | 0.023^***^ | 0.023^***^ |
|  | (0.012) | (0.012) | (0.008) | (0.008) |
|  |  |  |  |  |
| Ratio of core | -0.075^*^ | -0.075^*^ | -0.031 | -0.031 |
|  | (0.041) | (0.041) | (0.027) | (0.027) |
|  |  |  |  |  |
| Number of countries | -0.004 | -0.004 | -0.004^***^ | -0.004^***^ |
|  | (0.002) | (0.002) | (0.001) | (0.001) |
|  |  |  |  |  |
|  | | | | |
| Observations | 4,011 | 4,011 | 4,001 | 4,001 |
| R^2^ | 0.080 | 0.080 | 0.108 | 0.108 |
| Adjusted R^2^ | 0.073 | 0.073 | 0.101 | 0.101 |
|  | | | | |
| Note: | ^*^p^**^p^***^p<0.01 | | | |

**Table S22. Predicting *Creativity* with *Combined Inclusion*.** Table shows key variables and main controls (except year and platform dummies). Model 1) Baseline Model with standardized variables Model 2) Developer firms Random-Effects 3) Developer firms with Fixed-effects (as dummies) 4) Developer firm level aggregated models.

|  | Distinctiveness | | | |
| --- | --- | --- | --- | --- |
|  | *OLS Models* | | | |
|  | Std rob SE | Dev firm RE | Dev firm FE dummies | Dev firm level std |
|  | (1) | (2) | (3) | (4) |
| Diversity | 0.040^*^ | 0.033 | 0.032 | 0.043 |
|  | (0.021) | (0.022) | (0.025) | (0.038) |
| Combined Inclusion | -0.115^**^ | -0.137^**^ | -0.136^**^ | -0.173^*^ |
|  | (0.051) | (0.054) | (0.061) | (0.097) |
| Diversity:   Combined Inclusion | 0.048^***^ | 0.049^***^ | 0.032^*^ | 0.073^***^ |
|  | (0.016) | (0.017) | (0.019) | (0.028) |
| Team Size | - 0.001^***^ | -0.001^***^ | -0.001^***^ | -0.001^**^ |
|  | (0.0002) | (0.0002) | (0.0003) | (0.0004) |
| Newbies ratio | 0.158 | 0.149 | 0.179 | 0.082 |
|  | (0.115) | (0.114) | (0.134) | (0.238) |
| Games tenure | 0.038^*^ | 0.009 | 0.008 | 0.025 |
|  | (0.023) | (0.024) | (0.033) | (0.044) |
| Star developer | 0.135 | 0.305 | 0.340 | -0.349 |
|  | (0.310) | (0.382) | (0.306) | (0.239) |
| Single-firm production | 0.050 | 0.006 | -0.003 | -0.021 |
|  | (0.034) | (0.038) | (0.052) | (0.069) |
| Ratio of core | 0.200^**^ | 0.210^**^ | 0.230^**^ | 0.384 |
|  | (0.100) | (0.096) | (0.114) | (0.235) |
| Number of countries | -0.033^***^ | -0.029^***^ | -0.024^***^ | -0.040^***^ |
|  | (0.006) | (0.005) | (0.007) | (0.014) |
| N | 4,011 | 4,011 | 3,368 | 1,354 |
| R^2^ | 0.112 | 0.225 | 0.475 | 0.165 |
| Adjusted R^2^ | 0.105 | 0.219 | 0.327 | 0.146 |
| *Note:* | ^*^p<0.1; ^**^p<0.05; ^***^p<0.0 | | | |

**Table S23. Predicting Creativity as *Distinctivness* within various time ranges, 1,3,5,7 years with *Combined Inclusion***. Table shows key variables and main controls (except year and platform dummies). Model shows standardized variables and Robust SE.

|  | Distinctiveness | | | |
| --- | --- | --- | --- | --- |
|  |  | | | |
|  | 1 year | 3 years | 5 years | 7 years |
|  | 1yr rob SE | 3yr rob SE | 5yr rob SE | 7yr rob SE |
|  | (1) | (2) | (3) | (4) |
|  | | | | |
| Diversity | 0.025^*^ | 0.027^*^ | 0.027^*^ | 0.026^*^ |
|  | (0.014) | (0.014) | (0.014) | (0.014) |
|  |  |  |  |  |
| Combined | -0.001^**^ | -0.001^**^ | -0.001^**^ | -0.0005^**^ |
|  | (0.0002) | (0.0002) | (0.0002) | (0.0002) |
|  |  |  |  |  |
| Diversity:Combined | 0.002^***^ | 0.002^***^ | 0.002^***^ | 0.002^***^ |
|  | (0.001) | (0.001) | (0.001) | (0.001) |
| Team Size | -0.0001^***^ | -0.0001^***^ | -0.0001^***^ | -0.0001^***^ |
|  | (0.00001) | (0.00001) | (0.00001) | (0.00001) |
|  |  |  |  |  |
| Newbies ratio | 0.007 | 0.008 | 0.007 | 0.006 |
|  | (0.008) | (0.007) | (0.007) | (0.007) |
|  |  |  |  |  |
| Games Tenure | 0.002 | 0.002 | 0.002 | 0.002 |
|  | (0.002) | (0.001) | (0.001) | (0.001) |
|  |  |  |  |  |
| Star developer | 0.006 | 0.004 | 0.004 | 0.002 |
|  | (0.020) | (0.020) | (0.020) | (0.020) |
|  |  |  |  |  |
| Single-firm production | 0.002 | 0.002 | 0.003 | 0.003 |
|  | (0.002) | (0.002) | (0.002) | (0.002) |
|  |  |  |  |  |
| Ratio of core | 0.012^*^ | 0.012^*^ | 0.012^*^ | 0.012^*^ |
|  | (0.007) | (0.007) | (0.006) | (0.006) |
|  |  |  |  |  |
| Number of countries | -0.002^***^ | -0.002^***^ | -0.002^***^ | -0.002^***^ |
|  | (0.0004) | (0.0004) | (0.0004) | (0.0004) |
|  |  |  |  |  |
|  |  |  |  |  |
|  | | | | |
| Observations | 4,011 | 4,011 | 4,011 | 4,011 |
| R^2^ | 0.113 | 0.112 | 0.107 | 0.100 |
| Adjusted R^2^ | 0.106 | 0.105 | 0.100 | 0.093 |
|  | | | | |
| Note: | ^*^p^**^p^***^p<0.01 | | | |

**Table S24. Predicting *Creativity* with *Combined Inclusion* by genre category groups**. First group includes Action, adventure games, second Simulation, sport, and strategy. Table shows key variables and main controls (except year and platform dummies).

|  | Distinctiveness | |
| --- | --- | --- |
|  |  | |
|  | Action, adventure | Simulation, sports, strategy |
|  | (1) | (2) |
|  | | |
| Diversity | 0.054^***^ | 0.048^**^ |
|  | (0.015) | (0.019) |
|  |  |  |
| Combined | -0.001^***^ | -0.0005 |
|  | (0.0002) | (0.0003) |
| Diversity:Combined | 0.002^***^ | 0.002^**^ |
|  | (0.001) | (0.001) |
|  |  |  |
| Team Size | -0.00002 | -0.0001^***^ |
|  | (0.00001) | (0.00002) |
|  |  |  |
| Newbies ratio | -0.004 | 0.022^**^ |
|  | (0.008) | (0.010) |
|  |  |  |
| Games Tenure | -0.004^***^ | 0.007^***^ |
|  | (0.002) | (0.002) |
|  |  |  |
| Star developer | 0.023 | -0.020 |
|  | (0.022) | (0.027) |
|  |  |  |
| Single-firm production | 0.002 | 0.003 |
|  | (0.002) | (0.003) |
|  |  |  |
| Ratio of core | 0.015^**^ | 0.004 |
|  | (0.008) | (0.009) |
|  |  |  |
| Number of countries | -0.001^***^ | -0.002^***^ |
|  | (0.0005) | (0.001) |
|  |  |  |
|  |  |  |
|  | | |
| Observations | 2,381 | 2,371 |
| R^2^ | 0.149 | 0.134 |
| Adjusted R^2^ | 0.138 | 0.122 |
|  | | |
| Note: | ^*^p^**^p^***^p<0.01 | |

**Table S25. Predicting *Creativity* with *Combined Inclusion* by production company size**. Production company size is defined by the number of games a production firm has published within our database. First group includes games that was published by the Top 250 most publishing companies, second group includes games belonging to the Bottom 250. Table shows key variables and main controls (except year and platform dummies).

|  | | |
| --- | --- | --- |
|  | Distinctiveness | |
|  |  | |
|  | Top 1-250 | Top 251-smallest |
|  | (1) | (2) |
|  | | |
| Diversity | 0.036^*^ | 0.027 |
|  | (0.021) | (0.021) |
|  |  |  |
| Combined | -0.001^*^ | -0.0005 |
|  | (0.0003) | (0.0003) |
| Diversity:Combined | 0.002^*^ | 0.002^**^ |
|  | (0.001) | (0.001) |
|  |  |  |
| Team Size | -0.0001^***^ | -0.0001^***^ |
|  | (0.00002) | (0.00002) |
|  |  |  |
| Newbies ratio | 0.006 | 0.017 |
|  | (0.012) | (0.011) |
|  |  |  |
| Games Tenure | 0.004^*^ | 0.002 |
|  | (0.002) | (0.002) |
|  |  |  |
| Star developer | 0.029 | -0.066 |
|  | (0.026) | (0.047) |
|  |  |  |
| Single-firm production | 0.007^**^ | -0.003 |
|  | (0.003) | (0.004) |
|  |  |  |
| Ratio of core | 0.012 | 0.019^*^ |
|  | (0.010) | (0.011) |
|  |  |  |
| Number of countries | -0.003^***^ | -0.002^**^ |
|  | (0.001) | (0.001) |
|  |  |  |
|  |  |  |
|  | | |
| Observations | 2,186 | 1,825 |
| R^2^ | 0.108 | 0.135 |
| Adjusted R^2^ | 0.095 | 0.120 |
|  | | |
| Note: | ^*^p^**^p^***^p<0.01 | |

**Table S26. Predicting *Creativity* with *Combined Inclusion* by production year category groups.** First group includes games published between 1994-2001 second group includes 2001-2009. Table shows key variables and main controls (except year and platform dummies).

|  | | |
| --- | --- | --- |
|  | Distinctiveness | |
|  |  | |
|  | 1994-2001 | 2002-2009 |
|  | (1) | (2) |
|  | | |
| Diversity | 0.036^*^ | 0.021 |
|  | (0.020) | (0.022) |
|  |  |  |
| Combined | -0.001^*^ | -0.001^*^ |
|  | (0.0003) | (0.0004) |
| Diversity:Combined | 0.003^***^ | 0.002^*^ |
|  | (0.001) | (0.001) |
|  |  |  |
| Team Size | -0.00004^**^ | -0.0001^***^ |
|  | (0.00002) | (0.00002) |
|  |  |  |
| Newbies ratio | 0.004 | 0.023^*^ |
|  | (0.011) | (0.012) |
|  |  |  |
| Games Tenure | 0.004 | 0.002 |
|  | (0.003) | (0.002) |
|  |  |  |
| Star developer |  | 0.011 |
|  |  | (0.023) |
|  |  |  |
| Single-firm production | 0.005 | 0.001 |
|  | (0.003) | (0.004) |
|  |  |  |
| Ratio of core | -0.0002 | 0.029^***^ |
|  | (0.010) | (0.010) |
|  |  |  |
| Number of countries | -0.004^***^ | -0.002^***^ |
|  | (0.001) | (0.001) |
|  |  |  |
|  |  |  |
|  | | |
| Observations | 2,137 | 1,874 |
| R^2^ | 0.083 | 0.123 |
| Adjusted R^2^ | 0.073 | 0.113 |
|  | | |
| Note: | ^*^p^**^p^***^p<0.01 | |

**Table S27. Predicting *Novelty* as the log number of newly introduced style-pairings and the ratio of newly introduced style-pairings from all style-pairings based on Hofstra et al, 2020, Yang et al 2022 with *Combined Inclusion*.** Table shows key variables and main controls (except year and platform dummies). Model 1) Baseline Model with standardized variables and Robust SE Model 2) Baseline Model with standardized variables and Clustered SE.

|  | | | | |
| --- | --- | --- | --- | --- |
|  | Novelty | | | |
|  |  | | | |
|  | log10(Number of new pairs + 1) | | Ratio of new pairs | |
|  | rob SE | clus SE | rob SE | clus SE |
|  | (1) | (2) | (3) | (4) |
|  | | | | |
| Diversity | 0.065 | 0.065 | 0.041 | 0.041 |
|  | (0.075) | (0.075) | (0.049) | (0.049) |
|  |  |  |  |  |
| Combined | -0.002 | -0.002 | -0.002^**^ | -0.002^**^ |
|  | (0.001) | (0.001) | (0.001) | (0.001) |
| Diversity:Combined | 0.006^*^ | 0.006^*^ | 0.007^***^ | 0.007^***^ |
|  | (0.004) | (0.004) | (0.002) | (0.002) |
|  |  |  |  |  |
| Team Size | -0.00002 | -0.00002 | -0.0002^***^ | -0.0002^***^ |
|  | (0.0001) | (0.0001) | (0.00004) | (0.00004) |
|  |  |  |  |  |
| Newbies ratio | -0.017 | -0.017 | -0.017 | -0.017 |
|  | (0.041) | (0.041) | (0.027) | (0.027) |
|  |  |  |  |  |
| Games Tenure | -0.013^*^ | -0.013^*^ | -0.010^**^ | -0.010^**^ |
|  | (0.008) | (0.008) | (0.005) | (0.005) |
|  |  |  |  |  |
| Star developer | 0.074 | 0.074 | 0.026 | 0.026 |
|  | (0.154) | (0.154) | (0.058) | (0.058) |
|  |  |  |  |  |
| Single-firm production | 0.033^***^ | 0.033^***^ | 0.023^***^ | 0.023^***^ |
|  | (0.012) | (0.012) | (0.008) | (0.008) |
|  |  |  |  |  |
| Ratio of core | -0.022 | -0.022 | -0.015 | -0.015 |
|  | (0.035) | (0.035) | (0.023) | (0.023) |
|  |  |  |  |  |
| Number of countries | -0.004 | -0.004 | -0.004^***^ | -0.004^***^ |
|  | (0.002) | (0.002) | (0.001) | (0.001) |
|  |  |  |  |  |
|  |  |  |  |  |
|  | | | | |
| Observations | 4,011 | 4,011 | 4,001 | 4,001 |
| R^2^ | 0.080 | 0.080 | 0.110 | 0.110 |
| Adjusted R^2^ | 0.073 | 0.073 | 0.103 | 0.103 |
|  | | | | |
| Note: | ^*^p^**^p^***^p<0.01 | | | |

**Table S28. Predicting Game Review Score, with Heckman outcome model and OLS model**. Table shows key variables and main controls (except year and platform dummies).

|  | Review Score | | |
| --- | --- | --- | --- |
|  | *Heckman* | | *OLS* |
|  | Selection | Outcome | Ols |
|  | 1 | 2 | 3 |
| Diversity | -2.041^***^ |  |  |
|  | (0.290) |  |  |
| Distinctiveness |  | 0.974^***^ | 0.838^***^ |
|  |  | (0.133) | (0.135) |
| Diversity:  Combined Inclusion | | -0.012 | -0.036^***^ |
|  |  | (0.012) | (0.012) |
| Team Size | 0.008^***^ | 0.0003^**^ | 0.001^***^ |
|  | (0.001) | (0.0001) | (0.0001) |
| Games tenure |  | 0.021^**^ | 0.024^**^ |
|  |  | (0.010) | (0.010) |
| Star developer | 0.820 | 0.118 | 0.208 |
|  | (0.590) | (0.174) | (0.164) |
| Number of countries | 0.284^***^ |  |  |
|  | (0.019) |  |  |
| Single-firm production |  | 0.134^***^ | 0.120^***^ |
|  |  | (0.020) | (0.020) |
| Ratio of core |  | 0.188^***^ | 0.201^***^ |
|  |  | (0.057) | (0.059) |
| Controls added | No | No | No |
| Observations | 4,011 | 4,011 | 2,677 |
| R^2^ | 0.119 | 0.119 | 0.095 |
| Adjusted R^2^ | 0.111 | 0.111 | 0.088 |
| rho | -0.633 | -0.633 |  |
| Inverse Mills Ratio | -0.312^***^ (0.038) | -0.312^***^ (0.038) | |
| *Note:* | ^*^p<0.1; ^**^p<0.05; ^***^p<0.0 | | |

**Table S29.** List of publicly available employees including game reviewers at Kotaku (https://kotaku.com/)

| Kotaku | | |
| --- | --- | --- |
| Position | Name | Gender |
| editor-in-chief | Stephen Totilo | Male |
| deputy editor | Maddy Myers | Female |
| editor-at-large | Riley MacLeod | Male |
| news editor | Jason Schreier | Male |
| features editor | Chris Kohler | Male |
| senior editor | Natalie Degraffinried | Female |
| senior writer (nights) | Brian Ashcraft | Male |
| senior writer (nights) | Luke Plunkett | Male |
| senior reporter | Michael Fahey | Male |
| senior reporter | Nathan Grayson | Male |
| senior writer | Heather Alexandra | Female |
| staff writer | Ethan Gach | Male |
| staff writer | Ari Notis | Male |
| staff writer | Ian Walker | Male |
| weekend editor | Zack Zwiezen | Male |
| senior video producer | Chris Person | Male |
| video producer | Paul Tamayo | Male |
| contributor | GB Burford | Unknown |
| contributor | Kevin Wong | Male |
| contributor | Joshua Calixto | Male |
| contributor | S.E. Doster | Unknown |
| contributor | Lee Yancy | Unknown |
| contributor | Kate Gray | Female |
| contributor | Harris O’Malley | Male |
| contributor | Kirk Hamilton | Male |
| art director | Jim Cooke | Male |
| staff illustrator | Angelica Alzona | Female |
| staff illustrator | Chelsea Beck | Female |
| staff illustrator | Elena Scotti | Female |

**Table S30.** List of publicly available employees including game reviewers at Gaminformer (https://www.gameinformer.com/)

| Gaminformer | | |
| --- | --- | --- |
| Position | Name | Gender |
| editor-in-chief | Andy McNamara | Male |
| executive editor | Andrew Reine | Male |
| senior reviews editor | Joe Jubel | Male |
| senior previews editor | Matt Miller | Male |
| digital editor | Brian Shea | Male |
| pc editor | Daniel Tack | Male |
| features editor | Kimberley Wallace | Female |
| senior editor | Matthew Kato | Male |
| senior editor | Jeff Cork | Male |
| senior editor | Ben Reeves | Male |
| video producer | Leo Vader | Male |
| video editor | Alex Stadnik | Male |
| advertising manager | Janey Stringer | Female |
| marketing coordinator | Rachel Castle | Female |
| circulation services | Ted Katzung | Male |
| fulfillment specialist | Michelle Biros | Female |
| office manager | Sarah Hansen | Female |
| creative director | Jeff Akervik | Male |
| senior production director | Curtis Fung | Male |
| senior graphic designer | Laleh Tobin | Male |
| graphic designer | Jen Vinson | Female |
| web designer/programmer | Margaret Andrews | Female |
| web designer/programmer | Kristin Williams | Female |
| software engineer | Shawn Gilligan | Male |

**Table S31.** List of publicly available employees including game reviewers at Eurogamer (https://www.eurogamer.net/reviews)

| Eurogamer | | |
| --- | --- | --- |
| Position | Name | Gender |
| editor | Oli Welsh | Male |
| deputy editor | Wesley Yin-Poole | Male |
| feature and reviews editor | Martin Robinson | Male |
| news editor | Tom Phillips | Male |
| features editor | Christian Donlan | Male |
| guides editor | Matthew Reynolds | Male |
| senior staff writer | Robert Purchese | Male |
| staff writer | Chris Tapsell | Male |
| reporter | Matt Wales | Male |
| reporter | Emma Kent | Female |
| video team | Ian Higton | Male |
| video team | Johnny Chiodini | Male |
| video team | Aoife Wilson | Female |
| technology editor, digital foundry | Richard Leadbetter | Male |
| senior staff writer, digital foundry | Tom Morgan | Male |
| staff writer, digital foundry | John Linneman | Male |
| audience development director | Jon Hicks | Male |

Data S1. (available online)

Csv file used in analysis with all calculated variables on game level available here: <https://github.com/velf/moby_data>

Code S1. (available online)

Codebase used in analysis available here: <https://github.com/velf/moby_data>
